# Dynamic control of nonlinear emission by exciton-photon coupling in WS_2_ metasurfaces

**DOI:** 10.1126/sciadv.ady2108

**Published:** 2025-08-29

**Authors:** Mudassar Nauman, Domenico de Ceglia, Jingshi Yan, Lujun Huang, Mohsen Rahmani, Costantino De Angelis, Andrey E. Miroshnichenko, Yuerui Lu, Dragomir Neshev

**Affiliations:** ^1^ School of Engineering, The Australian National University, Canberra, ACT 2601, Australia.; ^2^ ARC Centre of Excellence for Transformative Meta-Optical Systems (TMOS), Department of Electronic Materials Engineering, Research School of Physics, The Australian National University, Canberra, ACT 2601, Australia.; ^3^ BluGlass Ltd., Sydney, NSW 2128, Australia.; ^4^ Department of Information Engineering, University of Brescia, Via Branze 38, 25123 Brescia, Italy.; ^5^ School of Engineering and Information Technology, University of New South Wales, Canberra, ACT 2600, Australia.; ^6^ Advanced Optics and Photonics Laboratory, Department of Engineering, School of Science and Technology, Nottingham Trent University, Nottingham NG11 8NS, UK.

## Abstract

Transition metal dichalcogenides are promising quantum materials because of unique exciton-photon interactions. These interactions can be enhanced by coupling with resonant photonic structures, especially in the nonlinear light emission processes like second-harmonic generation (SHG). However, excitonic absorption may dampen SHG. Here, we demonstrate tunable SHG enhancement using virtual coupling effects between quasi-bound state in the continuum (qBIC) optical resonances and tunable excitons in high-index WS_2_ metasurfaces with crescent meta-atoms. These metasurfaces promote a magnetic-type qBIC resonance, enabling control over nonlinear optical processes in the visible spectrum. The used qBIC resonance at half the exciton energy increases SHG efficiency by 98-fold compared to monolayer WS_2_ and by four orders of magnitude relative to an unpatterned WS_2_ film. The enhancement is tunable with temperature and incident light polarization, allowing the dynamic control of virtual coupling and SHG efficiency, thereby paving the way for next-generation reconfigurable metaoptics devices.

## Teaser

Virtual quantum-level exciton-photon interactions enabled efficient tunable nonlinear emissions.

## INTRODUCTION

Strong nonlinearities at the nanoscale, such as in subwavelength structures and metasurfaces (*1*–*4*), are of paramount importance for applications in nonlinear and quantum optics (*5*–*8*). Low-loss, high-index dielectric metasurfaces made of Si (*9*), Ge (*10*), and III-V semiconductors (*11*, *12*) are often the materials of choice for enhancing the nonlinear response at the nanoscale. However, most of the demonstrations reported to date have been focused on passive devices (*7*–*16*). Following the current need for industrial applications such as dynamic nonlinear holography, imaging (*13*), bioelectronics (*17*), and sensing (*18*), the new paradigm in the field of dielectric metasurfaces is to switch to active/tunable metasurfaces (*19*–*21*) and trigger the dynamic control of the nonlinear emission.

Transition metal dichalcogenides (TMDCs) offer an important alternative to conventional semiconductors because of their unique properties, such as high refractive index, novel weak van der Waals (vdWs) stiction forces, and atomically confined excitons even in bulk films (*22*–*24*). These properties offer new opportunities for the realization of tunable dielectric metasurfaces. For example, first, the high refractive index and weak vdWs stiction forces can be exploited to transform a flat bulk TMDC film into a deep subwavelength array of meta-atoms on any desired transparent substrate. Second, via external stimuli such as the environmental index (*25*), strain (*26*), electrical gating (*27*), and electrical and temperature treatment (*28*, *29*), the atomically confined excitons can be spectrally tuned over large spectral widths. In TMDCs, excitons exhibit exceptionally large binding energies, enabling their stability at room temperature (RT) and even higher temperature, in stark contrast to III-V semiconductors where excitons are only stable at cryogenic temperatures. The interaction of these excitons with light can be further enhanced by the presence of photonic resonances, such as in optical metasurfaces. The higher the quality factor (*Q*-factor) of the resonances, the stronger the exciton-photon coupling is. As such, metasurfaces exhibiting qBIC resonances have attracted strong recent interest (*5*, *13*, *30*), including for enhancing nonlinear emission (*5*). To date, however, the tunable nature of excitons has been exploited mainly in atomically thin TMDC films to realize tunable linear (*26*, *27*) and nonlinear (*28*, *31*, *32*) photonic devices. The few works exploiting bulk excitons have focused on the strong coupling regime, where the incident light is fully cycled between the excitons and the cavity, resulting in mode splitting (*33*–*35*). However, the absorption of the incident light is detrimental to nonlinear processes, such as the upconversion in the SHG process. To avoid the single-photon absorption of the excitation, the excitonic resonance should not be at the same energy as the qBIC resonance. To date, this regime has never been exploited in TMDC metasurfaces for the tunability and enhancement of nonlinear emissions. This research gap and the abovementioned findings raise the intriguing question of whether excitonic resonances in bulk TMDCs can be harnessed to underpin next-generation reconfigurable flat photonic devices.

In this work, we propose and demonstrate a tunable single-crystalline homogeneous subwavelength WS_2_ metasurface that supports high–*Q*-factor qBIC resonance at a fundamental wavelength of 1220 nm. As the bulk WS_2_ has excitonic resonance in the visible spectrum around 610 nm. Hence, a metasurface with qBIC at 1220 nm gives us an exciting opportunity to engineer the emergent physics (at the quantum level) between the exciton (610 nm) and second-harmonic (SH) photons of qBIC at RT. Such a strong excitonic-photonic quantum interaction in the WS_2_ metasurface enormously enhances the SHG by more than 98-fold compared to monolayer WS_2_ (1L-WS_2_) and four orders of magnitude compared to an unpatterned WS_2_ film. Intriguingly, the SHG enhancement can be tuned through external stimuli, such as temperature [which regulates A-type exciton ($E_{0}^{A}$)] and pump polarization angle (φ_pump_), which controls the qBIC excitation. Moreover, experimental studies and theoretical modeling have been done, and remarkable SHG enhancement is observed in the visible spectrum (600 to 650 nm), where WS_2_ is opaque. Our design opens previously unidentified strategies for highly tunable single-crystalline nonlinear photonic devices.

## RESULTS

### Design of the qBIC WS_2_ metasurface for tunable SHG

**Fig. 1.**
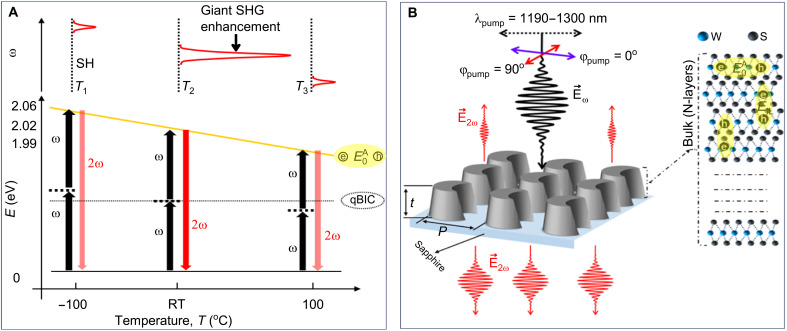
Principle of tunable significant enhancement in SHG. (**A**) Three-level model showing the thermally tunable energy level of exciton and its overlapping with the SH of qBIC. (**B**) Schematic illustration of the nonlinear WS_2_ qBIC metasurface having qBIC at 1220 nm (half the energy level of A-type exciton $E_{0}^{A}$) and the parameters *P* (periodicity) and *t* (thickness). Bulk WS_2_ is a layered material, and each layer has confined $E_{0}^{A}$, as shown in the inset. ${\vec{\text{E}}}_{\omega }$ and ${\vec{\text{E}}}_{2\omega }$ represent the electric field vectors for fundamental and SH frequency, respectively. SHG enhancement tunable via pump polarization (φ_pump_), which controls the qBIC excitation.

In TMDCs, owing to their weak vdWs stiction forces between layers, many salient features of monolayer and bilayers continue to exist even in the bulk form (*36*). These include spin layer locking (*37*) and intralayer A-type excitons ($E_{0}^{A}$). The SHG in TMDCs is intrinsically enhanced when the incident or generated photons are in resonance with the exciton (*28*, *31*, *32*, *38*). However, it is beneficial for the exciton to be in resonance with the two-photon energy of the incident light to reduce the excitonic absorption of the strong pump. As the SHG process is quadratic to the incident power, strong photonic resonances can be further used to boost the SHG efficiency. In this way, the excitonic $E_{0}^{A}$ and photonic qBIC resonances can interfere through a virtual energy level, resulting in the multiplication of the total SHG enhancement. We note that because of the inversion symmetry in the bulk TMDCs, the SHG is emitted only from the top and bottom layers (*39*), which can interfere in the far field. Intriguingly, likewise, in the monolayer form, the energy level of $E_{0}^{A}$ in bulk can be tuned as a function of a gated electric field (*28*) or temperature (*27*, *32*), thereby opening a unique avenue to engineer the position and strength of the SHG generation in TMDCs. The concept of this tunable spectral and energy enhancement is schematically depicted in the three-level energy diagram in Fig. 1A.

In our work, we chose WS_2_ as a bulk TMDC metasurface because of the convenience of spectrally matching the WS_2_ excitonic resonance with the available femtosecond (fs) lasers. However, the concept is applicable to other TMDC materials. In the proposed scheme, at RT, the qBIC and $E_{0}^{A}$ of the bulk WS_2_ metasurface are in resonance with the fundamental and SH waves simultaneously to enormously boost the SHG from the WS_2_ metasurface. Meanwhile, the change in temperature (from −100° to 100°C) spectrally tunes the $E_{0}^{A}$ (from 600 to 625 nm corresponding to energy levels of 2.06 to 1.99 eV, respectively), as shown in Fig. 1A. Such a spectral shift of $E_{0}^{A}$ (as a function of temperature) breaks the resonance condition, thereby significantly reducing the SHG. Because of the low thermo-optic coefficient of WS_2_, the change in temperature (from −100° to 100°C) does not alter the spectral position of qBIC. This property gives us a degree of freedom to manipulate the SHG by spectrally tuning only the $E_{0}^{A}$ excitonic energy near the SH wavelength as a function of temperature.

Along with temperature tunability, we consider pump polarization and wavelength-dependent SHG from the WS_2_ qBIC metasurface, as shown in Fig. 1B. The proposed metasurface has a thickness of *t* = 220 nm, a periodicity of *P* = 600 nm, and bottom and top radii of *r* = 220 and 180 nm, respectively. The parameters δ and δ″ shown in fig. S4 represent the radius and axial displacement of the cylindrical cavity carved into one side of each meta-atom. This modification breaks the metasurface symmetry and controls the coupling of the qBIC mode to free space radiation as a function of φ_pump_ at normal incidence (*40*). By tuning the pump wavelength, the SHG is enhanced proportionally to the square of the *Q*-factor of the qBIC resonance. However, a significant enhancement together with unidirectional emission of the SHG occurs when $\lambda_{\text{qBIC}}=2\times \lambda_{\text{exciton}}$ and φ_pump_ is along one of the major axes (φ_pump_ = 90°) of meta-atoms, as shown in Fig. 1B.

### Fabrication and linear response

**Fig. 2.**
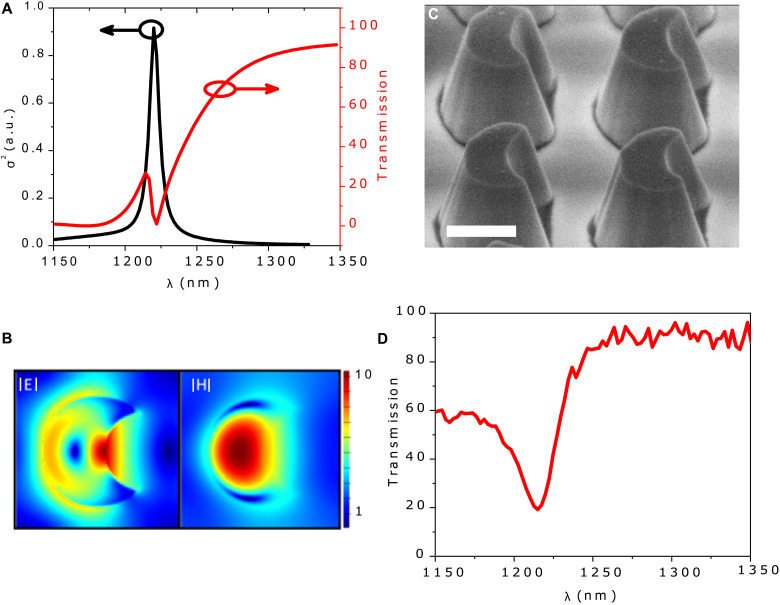
Experimental and numerical analysis of the WS_2_ metasurface. (**A**) Multipole expansion of metasurface A, where σ represents the effective cross section and simulated linear spectrum, showing qBIC resonance at 1220 nm. a.u., arbitrary units. (**B**) Calculated electric and magnetic field distributions at the resonance λ of 1220 nm. (**C**) SEM image of fabricated WS_2_ metasurface A on a sapphire substrate. This is our targeted metasurface because it has qBIC at 1220 nm (double the λ of $E_{0}^{A}$). The scale bar is 500 nm. (**D**) Measured linear spectrum of metasurface A.

To maintain the pristine quality, a large-area flat bulk WS_2_ film with a thickness of 220 nm has been mechanically exfoliated and dry transferred onto a sapphire substrate (~500 μm thick). Then, a flat bulk film is carved into subwavelength arrays of crescent meta-atoms using a standard electron beam lithography (EBL) method and dry etching. The detail of the fabrication method can be found in fig. S1. We patterned three metasurfaces, A to C, on the same flake, as illustrated in fig. S2. The three metasurfaces under study share identical geometric dimensions, except for variations in the central position of a cylindrical feature carved into one side of each meta-atom. This modification transforms the cone-shaped meta-atoms into crescent-shaped meta-atoms. In metasurface A, a cylindrical cavity with a radius of 150 nm and an axial displacement of 200 nm along the radial axis is introduced on one side of the cone-shaped meta-atoms, creating an asymmetric crescent shape, as depicted in Fig. 1B and fig. S4. This asymmetry induces a pure magnetic-type qBIC resonance, whose spectral position can be finely tuned by adjusting either the radius (δ) of the cylindrical cavity or its axial displacement (δ″). The three metasurfaces exhibit strong qBICs at 1220, 1270, and 1305 nm, respectively. As illustrated in Fig. 2A, a pure magnetic-type qBIC can be induced in the individual meta-atoms by suppressing the electric dipole (ED) through the excitation of an anapole mode (*41*) at the same spectral position as the magnetic dipole (MD).

The simulated linear transmission (red curve) and effective scattering cross section σ (black curve) of metasurface A (targeted metasurface), for which qBIC is at 1220 nm, are shown in Fig. 2A. The electric and magnetic field distributions are shown in Fig. 2B, demonstrating electric field circulation and magnetic field enhancement, as expected for a vertical MD mode. The radiation of this mode into the far field is controlled by the asymmetry parameter (δ″). Intriguingly, this asymmetry introduces polarization sensitivity (asymmetric coupling of qBIC resonance with φ_pump_) and enables tunable resonance properties for advanced optical applications, as discussed in detail in the Supplementary Materials (see the “qBIC excitation as a function of incident light polarization” section).

As the WS_2_ meta-atom is a nonmagnetic material, therefore, to identify the nature of induced qBIC, we performed multipole decomposition, as shown in Fig. 2A. The spherical ED has a zero response right at the spectral position where MD has the strongest response, as depicted in fig. S4. The zero response of spherical ED is a clear indication of the crossing point of Cartesian ED and toroidal dipole, resulting in the excitation of anapole mode at the MD resonance, “the pure MD scattering.” To enhance the second-order nonlinear response, this exotic ideal MD-type qBIC (MD qBIC) has not been exploited/excited so far in other recently reported nonmagnetic III-V semiconductor metasurfaces (*42*, *43*). The unfolding nature of the strongly enhanced magnetic field inside the resonator, as illustrated in Fig. 2B, further predicts the nature of qBIC (ideal MD), which is the most suitable candidate to strongly enhance the nonlinear light-matter interactions (*44*). In addition, in contrast to the magnetic field, the electric field strongly enhances outside the resonator into the carved volume, as shown in the left panel of Fig. 2B, making our metasurface suitable for hybrid structure applications, where strong coupling with another material is of paramount importance. In the proposed metasurface, the contribution of other higher-order modes, such as electric quadrupole and magnetic quadrupole, is negligible.

The scanning electron microscopy (SEM) image in Fig. 2C demonstrates the high quality of the fabricated metasurfaces. The optical image in fig. S2 delivers comprehensive details about the metasurface quality, as it also offers additional insights into uniformity by examining the color of the image and still resolves the nanostructuring. We used custom-built white light transmission spectroscopy to measure the linear transmission spectrum of metasurfaces A to C. Figure 2D displays the measured linear transmission spectrum of metasurface A. As shown in Fig. 2 (A and D), a close correspondence is evident between the measured and simulated transmission spectra.

### SHG in qBIC TMDC metasurfaces

To examine the enhancement factor (EF) of the SHG process in the qBIC TMDC metasurfaces, we used a tunable femtosecond laser (Chameleon Ultra II and OPO, pulse width of ~200 fs) in a home-built microscopy setup. Wavelength-dependent SHG measurements have been performed on each metasurface by focusing the laser onto the metasurfaces using a 5× microscope objective (numerical aperture, 0.3) to provide a broader illumination area for uniform excitation. Then, the SHG signal was collected in the forward direction using a 20× microscope objective (numerical aperture, 0.4), enhancing SHG signal collection and spatial resolution. A short-pass filter at 800 nm has been used to filter out the transmitted and reflected pump beams. In addition, two waveplates have been used to control the polarization of the excitation beam.

**Fig. 3.**
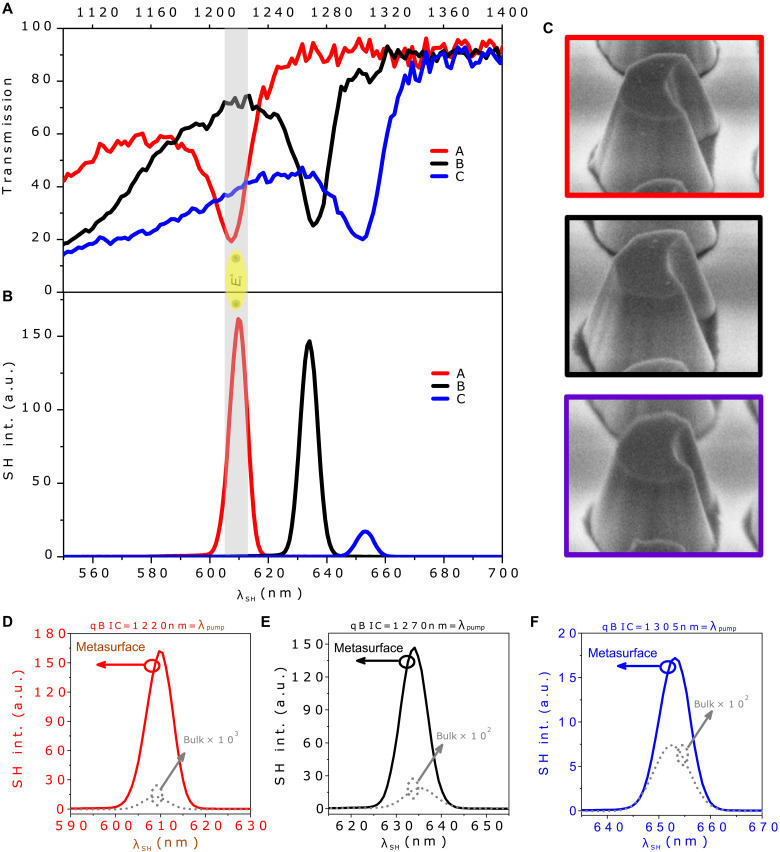
Measured SHG of MD qBIC WS_2_ metasurfaces. (**A**) Measured linear spectrum of metasurfaces A (MD qBIC: 1220 nm), B (MD qBIC: 1270 nm), and C (MD qBIC: 1305 nm). (**B**) Measured SHG of metasurfaces A to C. The light red–shaded area is the region where λqBIC:12202 falls at the spectral position of $E_{0}^{A}$ (610 nm), and the light black area is the region where λqBIC:12702 falls at the split resonance of $E_{0}^{A}$ ($E_{0}^{*A}$: 635 nm); this split appears in the multilayer structure owing to the strong coupling between Fabry-Perot modes and $E_{0}^{A}$ (*50*). Moreover, the light blue area is the region where λqBIC:13052 falls at the spectral position of 652 nm, and this area is far from $E_{0}^{A}$. (**C**) SEM images showing unit cells of metasurfaces A to C following the color-coding schemes used in (A). (**D** to **F**) Comparison of measured SHG enhancement between metasurfaces and the unpatterned WS_2_ film: (D) Metasurface A: The EF is 12,777. (E) Metasurface B: The EF is 7,850. (F) Metasurface C: The EF is 238.

The pristine WS_2_ bulk film is 2H type and has inversion symmetry, leading to intrinsically weak SHG. However, the presence of interfaces can break this symmetry. This results in a nonzero, albeit weak, surface-like SHG response at the material-air and material-substrate interfaces. However, TMDCs have high refractive index, pristine crystal quality, and huge anisotropic properties (*45*), which are crucial for enhancing the nonlinear response like SHG. To unlock this potential, we patterned pristine WS_2_ bulk film into metasurfaces A to C that support MD qBICs at pump wavelengths of 1220, 1270, and 1305 nm, respectively, as depicted in Fig. 3A. We used the above experimental setup and measured the wavelength-dependent SH response of our metasurfaces A to C. For this type of experiment, we tune the pump wavelength to the spectral positions of the MD qBICs at 1220, 1270, and 1305 nm in metasurfaces A to C, respectively. We have observed significant SH enhancement compared to the unpatterned WS_2_ film in all three metasurfaces when the pump wavelength tuned to the spectral positions of the MD qBICs supported by metasurfaces A to C (fig. S6).

Figure 3A shows the measured transmission spectra of the three WS_2_ metasurfaces A to C at RT. In the shaded region, metasurface A supporting the MD qBIC at 1220 nm (resonant at $\frac{\lambda_{\text{qBIC}}}{2}$) will interfere with the $E_{0}^{A}$ ($\lambda_{E_{0}^{A}}=610$ nm) of WS_2_ meta-atoms, thereby achieving enormously enhanced SHG, as shown in Fig. 3B. This is the region where metasurface A is under doubly resonant condition, namely $\lambda_{E_{0}^{A}}$ and harmonic λqBIC2 match or $\lambda_{\text{qBIC}}=2\times \lambda_{E_{0}^{A}}$, thereby significantly enhancing the SHG. Moreover, power-dependent SHG measurements confirm the nonlinear nature of the signal, exhibiting a quadratic dependence on pump power (fig. S7), as expected for a second-order process. For metasurface B, this enhanced SHG is reduced because the doubly resonant condition starts breaking. The SHG significantly reduced for metasurface C because λqBIC2 falls far from the $E_{0}^{A}$ spectral position, as shown in Fig. 3A. The spectral positions of MD qBIC resonances can be tuned by changing the asymmetry factor in WS_2_ arrays of meta-atoms, as shown by SEM images in Fig. 3C, following the color-coding schemes used in Fig. 3A.

In the shaded region, metasurface A has ninefold stronger SHG (owing to stronger MD qBIC-excitonic effects triggered by doubly resonant condition: $\lambda_{\text{qBIC}}=2\times \lambda_{E_{0}^{A}}$) compared to metasurface C, as illustrated in Fig. 3B. The EF of SHG in MD qBIC WS_2_ metasurfaces compared to the reminiscent bulk film (intentionally unpatterned film sitting next to metasurfaces) is presented in Fig. 3 (D to F). A microscopy image is introduced in fig. S2, showing the fabricated metasurfaces and unpatterned WS_2_ films.

The strong excitonic-photonic interference effect in metasurface A (enabled by doubly resonant condition between the MD qBIC and $E_{0}^{A}$) resulted in four orders of magnitude enhanced SHG compared to the unpatterned WS_2_ film at the same spectral position, as shown in Fig. 3D. Notably, the enhanced SHG from metasurface A is more than 98-fold stronger than 1L-WS_2_ [exfoliated from the same bulk crystals (HQ graphene, 2H-WS_2_) as we used in our metasurface] as discussed in detail in the Supplementary Materials (see the “SHG efficiency comparison of metasurface A with WS_2_ monolayer” section). Similarly, metasurface B, for which the MD qBIC is in the vicinity of the $E_{0}^{A}$ exciton, resulting in three orders of magnitude, enhanced SHG compared to the unpatterned WS_2_ film, as shown in Fig. 3E. For metasurface C, for which we have only MD qBIC resonance at a pump wavelength of 1305 nm and not meeting the doubly resonant criteria ($\lambda_{\text{qBIC}}\neq 2\times \lambda_{E_{0}^{A}}$), we observe only an enhancement of two orders of magnitude SHG compared to the unpatterned WS_2_ film, as shown in Fig. 3F. The large SHG enhancement in metasurface C arises from the high-*Q* MD qBIC resonance at 1305 nm. These modes confine light efficiently, boosting the local field intensity and nonlinear response (*4*, *5*). The high *Q*-factor prolongs the resonance lifetime, enhancing energy buildup and SHG by two orders of magnitude versus unpatterned WS_2_. This follows the expected quadratic scaling $I_{\text{SH}}\propto Q^{2}I_{o}^{2}$ (*40*). In a nutshell, the MD qBIC alone enhances two orders of magnitude SHG in TMDC metasurfaces (as in metasurface C), while this enhancement can be boosted to four orders of magnitude by exploiting the excitonic-photonic interference effects in metasurface A enabled by doubly resonant criteria ($\lambda_{\text{qBIC}}=2\times \lambda_{E_{0}^{A}}$), as shown in Fig. 3.

### Dynamic control on the SHG in TMDC metasurfaces

**Fig. 4.**
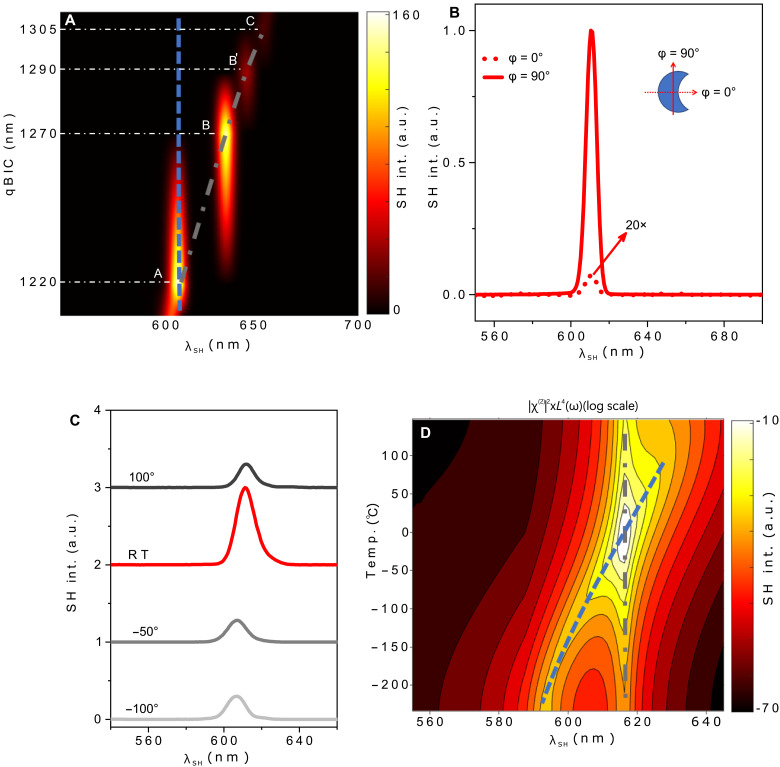
Measured and simulated tunable SHG of MD qBIC WS_2_ metasurfaces. (**A**) Measured SHG mapping as a function of MD qBIC and excitation wavelength. The spectral position of MD qBIC has been tuned by changing the δ″. The vertical dashed blue line shows the position of $E_{0}^{A}$ in bulk WS_2_, while the gray dash-dot line shows the spectral tuning of MD qBIC as a function of the parameter δ″. The horizontal dashed lines are the eye guide for the MD qBIC spectral positions of metasurfaces A, B, B′, and C. (**B**) Measured SHG enhancement of metasurface A when the pump polarization angles φ = 0° and φ = 90°, with the inset showing the schematic of φ of the incident light with respect to the crescent-shaped meta-atom geometry. (**C**) Measured wavelength-dependent SHG of metasurface A as a function of temperature with a temperature change of 100° to −100°C around RT. (**D**) Simulated wavelength-dependent SHG of metasurface A as a function of temperature ranging from 100° to −190°C. Results presented in (A) and (B) are measured under ambient conditions (RT).

Excitonic enhanced SHG has been previously reported in atomically thin TMDCs (*28*, *31*, *32*), and these excitonic effects may exist even in the bulk form (*24*), thereby opening opportunities to explore fundamental physics in TMDCs (*35*, *46*, *47*). Direct excitation of optical resonances at the excitonic resonance results in mode splitting (*22*, *35*), diverting energy from the pump and introducing a nonlinear loss mechanism, thereby suppressing SHG efficiency. To circumvent these limitations, we proposed a promising strategy to detune the MD qBIC from excitonic resonance and excite at double the excitonic wavelength ($\lambda_{\text{qBIC}}=2\times \lambda_{E_{0}^{A}}$). This approach leverages virtual (quantum level) exciton-qBIC interaction at RT and significantly enhances the SHG without resonant absorption losses. To dynamically control the SHG enhancement, this exciton-qBIC virtual interaction can be made tunable by three distinct approaches: by spectrally tuning the MD qBIC at the fundamental wavelength, by controlling/tuning the excitation of the MD qBIC at the fundamental wavelength, or by spectrally tuning the exciton at the SH wavelength. In the first approach, we spectrally tune the MD qBIC at the fundamental wavelength by changing the parameter δ″ of the fabricated metasurfaces (A, B, B′, and C) while keeping the other parameters constant, as illustrated in Fig. 4A. It can be observed in Fig. 4A that metasurface A supporting the MD qBIC at 1220 nm (meeting the doubly resonant condition: $\lambda_{\text{qBIC}}=2\times \lambda_{E_{0}^{A}}$) exhibits the strongest SHG. As shown in Fig. 4A, this SHG enhancement starts reducing as we start breaking the doubly resonant condition by tuning the MD qBIC resonance toward longer pump wavelengths (1270, 1290, and 1305 nm) by changing the parameter δ″ in metasurfaces B, B′, and C, respectively. To validate the experimental approach and assess the MD qBIC tunability effect on the exciton-qBIC doubly resonant condition and its related SHG enhancement, we performed full-wave nonlinear simulations, as detailed in the Supplementary Materials (see the “Spectral tuning of qBIC at pump wavelengths” section). To verify the experimental results reported in Fig. 4A, in our numerical simulations, we spectrally tune the MD qBIC at pump wavelengths (by changing the asymmetry) to break and restore the doubly resonant condition. It can be observed in Fig. 4A and simulated results shown in fig. S10 that SHG significantly enhances when the MD qBIC meets the doubly resonant condition. Both results are in good agreement.

Our second approach controls the MD qBIC excitation optically, simply by adjusting the incident light’s polarization angle [φ, also referred to as the pump polarization angle (φ_pump_) in this work]. As shown in Fig. 4B, the MD qBIC resonance in our WS_2_ metasurface is highly polarization dependent. For metasurface A, the MD qBIC at 1220 nm activates at φ = 90° ($\lambda_{\text{qBIC}}=2\times \lambda_{E_{0}^{A}}$) but turns off at φ = 0°. This switching leads to two orders of magnitude SHG enhancement when the MD qBIC is on, as shown in Fig. 4B. Compared to unpatterned WS_2_, metasurface A achieves four orders of magnitude SHG boost at φ = 90°, as depicted in Fig. 3D, making polarization a powerful tuning knob for SHG at RT. Polarization-resolved SHG measurements (φ = 0° to 360°) reveal a dipole-like pattern (fig. S9), contrasting with bulk WS_2_’s sixfold symmetry. This shows that our MD qBIC metasurface can optically reshape SHG emission—a unique feature controllable purely by φ.

The final approach uses spectral tuning of the exciton $E_{0}^{A}$ at SH wavelengths by leveraging some external influences like temperature to align with the desired state. It is noteworthy that this tunability can also be achieved via other external stimuli, such as strain (*26*) or electric field (*28*), but temperature was the accessible method in our current experimental setup. In our work, to clearly indicate the SHG EF at RT in comparison to the other temperatures, we have considered a small temperature change of −100° to 100°C around RT, and such a temperature range can spectrally tune $E_{0}^{A}$ at SH wavelengths (from 600 to 622 nm) to break and/or restore the doubly resonant condition ($\lambda_{\text{qBIC}}=2\times \lambda_{E_{0}^{A}}$), as shown in Fig. 4C. To experimentally examine the temperature-dependent SHG behavior of our targeted metasurface A, we placed our sample in a Linkam chamber (attached to the temperature controller and liquid nitrogen), varied the temperature of our fabricated metasurface A, and then measured its wavelength-dependent SHG in the temperature range of −100° to 100°C with the following steps [−100°, −50°, (RT), and 100°C], as shown in Fig. 4C. It can be observed in Fig. 4C that the SHG at RT is almost 4.3× stronger than the SHG at other temperatures, i.e., −100°C. This is because, at RT, the $E_{0}^{A}$ and MD qBIC meet the doubly resonance condition: $\lambda_{\text{qBIC}}=2\times \lambda_{E_{0}^{A}}$. However, at other temperatures, the $E_{0}^{A}$ starts spectrally detuning from its RT spectral position (~610 nm) and exhibits a condition where $\lambda_{E_{0}^{A}}\neq \frac{\lambda_{\text{qBIC}}}{2}$. Owing to the wider nature of $E_{0}^{A}$ resonance in bulk WS_2_, this shift is not sufficient to completely break the doubly resonant condition but is enough to affect the SHG enhancement and observe its effect.

To verify our experimental results shown in Fig. 4C, we further performed full-wave numerical simulations to assess the temperature tunability of excitonic-photonic enhanced SHG from metasurface A, and results are illustrated in Fig. 4D. The details of numerical simulations related to the temperature tunability can be found in the Supplementary Materials (see the “Spectral tuning of exciton at SH wavelengths” section and figs. S11 to S13). In Fig. 4D, the blue dashed line is the eye guide for the spectral position of $E_{0}^{A}$, while the gray dash-dot line represents the spectral position of MD qBIC in the proposed WS_2_ metasurface A. It can be observed in Fig. 4D that variations in temperature relative to the RT spectrally detune the $E_{0}^{A}$ from its spectral position at RT (~610 nm). This spectral tuning of $E_{0}^{A}$ as a function of temperature can break and subsequently restore the doubly resonant condition ($\lambda_{\text{qBIC}}=2\times \lambda_{E_{0}^{A}}$) established at RT in metasurface A, thereby enabling dynamic control on the SHG enhancement as a function of an external stimulus, as illustrated in Fig. 4D. The measured and simulated results illustrated in Fig. 4 (C and D), respectively, are in good agreement and show that SHG enhances at RT when the $E_{0}^{A}$ and MD qBIC satisfy the doubly resonant condition ($\lambda_{\text{qBIC}}=2\times \lambda_{E_{0}^{A}}$). The underlying physics of the doubly resonant condition enhanced SHG along with its dependence on MD qBIC spectral position as a function of δ″ and external stimuli such as temperature can be comprehensively explained via the application of Fermi’s golden rule for SHG conversion efficiency (for details, see the “Fermi’s golden rule” section in the Supplementary Materials).

## DISCUSSION

In contrast to artificially stacked hybrid systems (a monolayer coupled to other dielectric metasurfaces) (*38*, *48*, *49*), bulk TMDCs naturally provide a cleaner system and an exciting opportunity to explore strong interactions between inelastic (excitonic properties) and elastic scattering properties (such as high-*Q* qBICs) (*35*, *50*, *51*). Owing to many exciting properties (*24*, *35*, *52*), bulk TMDCs offer the most promising avenue to date to subwavelength solid-state devices for tunable nonlinear optics (*39*). In this work, we have reported all-optically and thermally tunable significant SHG enhancement in single-crystalline qBIC WS_2_ metasurfaces. The significant SHG intensity enhancement in WS_2_ metasurfaces is the result of the simultaneous resonant conditions or doubly resonant criteria ($\lambda_{\text{qBIC}}=2\times \lambda_{E_{0}^{A}}$) between $E_{0}^{A}$ and the MD qBIC at SH and fundamental wavelengths, respectively.

To enhance the SH intensity, three approaches have been explored: spectral tuning of MD qBIC at pump wavelength as a function of the asymmetry parameter δ″, tuning excitation of induced MD qBIC at particular pump wavelength as a function of φ of the incident light, and spectral tuning of $E_{0}^{A}$ at SH wavelength as a function of temperature. In comparison to the change in geometrical parameters of metasurfaces, the last two approaches provide us easy platforms to manipulate the doubly resonant condition of $E_{0}^{A}$ and MD qBIC as a function of φ of the incident light and temperature to control the SHG in TMDC metasurfaces.

To engineer the doubly resonant condition in metasurface A, we controlled the excitation of MD qBIC at a pump wavelength of 1220 nm as a function of φ of the incident light. By just changing the φ of the incident light from 90° to 0°, we can activate or deactivate the MD qBIC resonance at a pump wavelength of 1220 nm to restore and/or break the doubly resonant condition in metasurface A. When the MD qBIC is activated φ = 90°, metasurface A exhibits two orders of magnitude SHG enhancement in comparison to the MD qBIC deactivation state φ = 0°. This all-optically controlled method also helped us to modulate the intrinsic sixfold SHG radiation pattern of the bulk WS_2_ metasurface into a dipole pattern. Moreover, we performed wavelength-dependent SHG at a temperature range of −100° to 100°C around RT to further engineer the doubly resonant condition in metasurface A. By increasing and decreasing the temperature, the spectral position of $E_{0}^{A}$ can be tuned toward longer and shorter wavelengths, respectively. Therefore, by changing the temperature, it can be observed that when the harmonic energy of the MD qBIC of metasurface A overlaps with the energy of $E_{0}^{A}$ (satisfying the doubly resonant condition), then a significant enhancement of four orders of magnitude in SHG can be observed compared to the unpatterned WS_2_ film. Under doubly resonant condition at RT, the proposed WS_2_ crescent metasurface A achieves SHG efficiency 98-fold (experimentally) and three orders of magnitude (numerically) stronger than that of 1L-WS_2_. The difference in experimental and simulated results is due to the difference in the measured and simulated *Q*-factors of the MD qBIC and local pump intensity. This substantial enhancement underscores the unique advantage of exploiting MD qBIC resonances along with the intrinsic excitonic resonances in bulk TMDC metasurfaces for nonlinear light-matter interactions. We also show the ninefold enhancement of metasurface A compared to metasurface C, for which the $E_{0}^{A}$ excitonic energy and the harmonic energy of the MD qBIC are not under doubly resonant condition. In metasurface A, the highest measured SHG efficiency is estimated to be about 5.8 × 10^−9^ at an incident peak power of 3.56 kW (fig. S15). The simulated SHG efficiency of metasurface A is three orders of magnitude stronger than the 1L-WS_2_ and five orders of magnitude stronger than the unpatterned WS_2_ (sharing the parent flake as of metasurfaces).

The enhanced SHG from our MD qBIC metasurfaces can be applied to a range of practical applications, including frequency conversion, sensing, and integrated photonic circuits (*21*, *53*, *54*). For frequency conversion, the significant SHG enhancement could enable more efficient generation of tunable wavelengths for telecom, spectroscopy, and biomedical imaging. In sensing, this enhanced SHG could improve optical sensor sensitivity and detection limits for nonlinear optical sensors, allowing for precise distance measurements with nanometer resolution, aiding in chemical, biological, and environmental sensing. In addition, our metasurfaces could be integrated into photonic circuits for on-chip frequency conversion, enabling more compact and efficient devices for quantum information processing. By detecting changes in the SHG signal, our metasurfaces can identify the presence of specific biomolecules, making them valuable for biomedical diagnostics. Localization of light via magnetic-type resonances in dielectric metasurfaces can suppress undesirable free carrier contribution, leading to ultrafast all-optical switching (*21*). Our metasurfaces support pure MD qBICs, thereby making them a valuable candidate for ultrafast all-optical switching applications. The proposed method not only has the potential for tunable SHG enhancement but also lay the foundation for extending this approach to advanced nonlinear optical processes, such as spontaneous parametric downconversion (*55*) for next-generation quantum applications. Last but not least, the proposed metasurface A achieves four orders of magnitude SHG enhancement over unpatterned bulk WS_2_ and enables 10^2^× all-optical modulation, both of which are critical for on-chip nonlinear optics.

We note that electrical tuning of the excitonic resonances can also be used to realize compact tunable devices. However, the mixing between exciton and trion resonances must be taken into account. Moreover, we also anticipate that applying the proposed doubly resonant approach ($\lambda_{\text{qBIC}}=2\times \lambda_{E_{0}^{A}}$) to high-efficiency materials such as 3R-MoS_2_ would further enhance the second-order nonlinear response and broaden the applicability. While our current work rigorously validates the concept by engineering the 2H-TMDCs (having surface-like nonlinearities), extending the method to 3R-MoS_2_ (naturally noncentrosymmetric: exhibiting bulk-like nonlinearities) is a promising next step.

## MATERIALS AND METHODS

### Device fabrication

We fabricated multiple arrays of WS_2_ crescent-shaped meta-atoms (WS_2_ metasurfaces) on a sapphire (Al_2_O_3_) substrate by using the EBL method (*56*, *57*). To ensure pristine sample quality, the WS_2_ vdWs material was mechanically exfoliated onto the substrate. The thickness of the transferred flakes was measured using a surface profilometer. Following exfoliation, the flakes were cleaned and coated with a single layer of ZEP positive resist, followed by a thin layer of conductive polymer to mitigate charging effects during EBL. The selected flake was patterned into crescent-shaped meta-atoms using EBL. Postdevelopment, a 60-nm-thick aluminum layer was deposited via electron-beam evaporation to serve as a dry etching mask. Crescent-shaped meta-atoms were then defined by dry etching using an inductively coupled plasma process with SF_6_, CHF_3_, and Ar gases. The aluminum caps atop the truncated-cone pillars were subsequently removed using a lift-off process. Last, the quality and dimensions of the WS_2_ metasurfaces were assessed through SEM.

## Supplementary Materials

### This PDF file includes:

Figs. S1 to S15 Supplementary Text References

## References

[1] M. V. Rybin, K. L. Koshelev, Z. F. Sadrieva, K. B. Samusev, A. A. Bogdanov, M. F. Limonov, Y. S. Kivshar, High-*Q* supercavity modes in subwavelength dielectric resonators. Phys. Rev. Lett. 119, 243901 (2017). 29286713 10.1103/PhysRevLett.119.243901

[2] K. Koshelev, G. Favraud, A. Bogdanov, Y. Kivshar, A. Fratalocchi, Nonradiating photonics with resonant dielectric nanostructures. Nanophotonics 8, 725–745 (2019).

[3] K. Sergey, Y. Kivshar, Functional meta-optics and nanophotonics governed by Mie resonances. ACS Photonics 4, 2638–2649 (2017).

[4] K. Koshelev, S. Lepeshov, M. Liu, A. Bogdanov, Y. Kivshar, Asymmetric metasurfaces with high-*Q* resonances governed by bound states in the continuum. Phys. Rev. Lett. 121, 193903 (2018). 30468599 10.1103/PhysRevLett.121.193903

[5] K. Koshelev, Y. Tang, K. Li, D.-Y. Choi, G. Li, Y. Kivshar, Nonlinear metasurfaces governed by bound states in the continuum. ACS Photonics 6, 1639–1644 (2019).

[6] K. Koshelev, A. Bogdanov, Y. Kivshar, Meta-optics and bound states in the continuum. Sci. Bull. 64, 836–842 (2019). 10.1016/j.scib.2018.12.003 36659673

[7] L. Carletti, S. S. Kruk, A. A. Bogdanov, C. De Angelis, Y. Kivshar, High-harmonic generation at the nanoscale boosted by bound states in the continuum. Phys. Rev. Res. 1, 023016 (2019).

[8] K. Koshelev, S. Kruk, E. Melik-Gaykazyan, J.-H. Choi, A. Bogdanov, H.-G. Park, Y. Kivshar, Subwavelength dielectric resonators for nonlinear nanophotonics. Science 367, 288–292 (2020). 31949078 10.1126/science.aaz3985

[9] C. Schlickriede, S. S. Kruk, L. Wang, B. Sain, Y. Kivshar, T. Zentgraf, Nonlinear imaging with all-dielectric metasurfaces. Nano Lett. 20, 4370–4376 (2020). 32374616 10.1021/acs.nanolett.0c01105

[10] G. Grinblat, Y. Li, M. P. Nielsen, R. F. Oulton, S. A. Maier, Efficient third harmonic generation and nonlinear subwavelength imaging at a higher-order anapole mode in a single germanium nanodisk. ACS Nano 11, 953–960 (2017). 27977932 10.1021/acsnano.6b07568

[11] S. Liu, M. B. Sinclair, S. Saravi, G. A. Keeler, Y. Yang, J. Reno, G. M. Peake, F. Setzpfandt, I. Staude, T. Pertsch, I. Brener, Resonantly enhanced second-harmonic generation using III-V semiconductor all-dielectric metasurfaces. Nano Lett. 16, 5426–5432 (2016). 27501472 10.1021/acs.nanolett.6b01816

[12] R. Camacho-Morales, M. Rahmani, S. Kruk, L. Wang, L. Xu, D. A. Smirnova, A. S. Solntsev, A. Miroshnichenko, H. H. Tan, F. Karouta, S. Naureen, K. Vora, L. Carletti, C. De Angelis, C. Jagadish, Y. S. Kivshar, D. N. Neshev, Nonlinear generation of vector beams from algaas nanoantennas. Nano Lett. 16, 7191–7197 (2016). 27797212 10.1021/acs.nanolett.6b03525

[13] F. Yesilkoy, E. R. Arvelo, Y. Jahani, M. Liu, A. Tittl, V. Cevher, Y. Kivshar, H. Altug, Ultrasensitive hyperspectral imaging and biodetection enabled by dielectric metasurfaces. Nat. Photonics 13, 390–396 (2019).

[14] V. Kravtsov, E. Khestanova, F. A. Benimetskiy, T. Ivanova, A. K. Samusev, I. S. Sinev, D. Pidgayko, A. M. Mozharov, I. S. Mukhin, M. S. Lozhkin, Y. V. Kapitonov, A. S. Brichkin, V. D. Kulakovskii, I. A. Shelykh, A. I. Tartakovskii, P. M. Walker, M. S. Skolnick, D. N. Krizhanovskii, I. V. Iorsh, Nonlinear polaritons in a monolayer semiconductor coupled to optical bound states in the continuum. Light Sci. Appl. 9, 56 (2020). 32284858 10.1038/s41377-020-0286-z PMC7145813

[15] P. Xie, Z. Liang, T. Jia, D. Li, Y. Chen, P. Chang, H. Zhang, W. Wang, Strong coupling between excitons in a two-dimensional atomic crystal and quasibound states in the continuum in a two-dimensional all-dielectric asymmetric metasurface. Phys. Rev. B 104, 125446 (2021).

[16] Y. Xie, Q. Chen, J. Yao, X. Liu, Z. Dong, J. Zhu, Dielectric metasurface evolution from bulk to monolayer by strong coupling of quasi-BICs for second harmonic boosting. Photonics Res. 12, 784–792 (2024).

[17] Z. Li, X. Tian, C.-W. Qiu, J. S. Ho, Metasurfaces for bioelectronics and healthcare. Nat. Electron. 4, 382–391 (2021).

[18] B. Schwarz, Mapping the world in 3D. Nat. Photonics 4, 429–430 (2010).

[19] J. Yu, S. Park, I. Hwang, D. Kim, F. Demmerle, G. Boehm, M. C. Amann, M. A. Belkin, J. Lee, Electrically tunable nonlinear polaritonic metasurface. Nat. Photonics 16, 72–78 (2022).

[20] A. Krasnok, M. Tymchenko, A. Alù, Nonlinear metasurfaces: A paradigm shift in nonlinear optics. Mater. Today 21, 8–21 (2018).

[21] G. Li, S. Zhang, T. Zentgraf, Nonlinear photonic metasurfaces. Nat. Rev. Mater. 2, 17010 (2017).

[22] T. Weber, L. Kühner, L. Sortino, A. Ben Mhenni, N. P. Wilson, J. Kühne, J. J. Finley, S. A. Maier, A. Tittl, Intrinsic strong light-matter coupling with self-hybridized bound states in the continuum in van der Waals metasurfaces. Nat. Mater. 22, 970–976 (2023). 37349392 10.1038/s41563-023-01580-7 PMC10390334

[23] S. Das, G. Gupta, K. Majumdar, Layer degree of freedom for excitons in transition metal dichalcogenides. Phys. Rev. B 99, 165411 (2019).

[24] A. Arora, M. Drüppel, R. Schmidt, T. Deilmann, R. Schneider, M. R. Molas, P. Marauhn, S. M. de Vasconcellos, M. Potemski, M. Rohlfing, R. Bratschitsch, Interlayer excitons in a bulk van der Waals semiconductor. Nat. Commun. 8, 639 (2017). 28935879 10.1038/s41467-017-00691-5 PMC5608874

[25] A. V. Stier, N. P. Wilson, G. Clark, X. Xu, S. A. Crooker, Probing the influence of dielectric environment on excitons in monolayer WSe_2_: Insight from high magnetic fields. Nano Lett. 16, 7054–7060 (2016). 27718588 10.1021/acs.nanolett.6b03276

[26] B. Aslan, M. Deng, T. F. Heinz, Strain tuning of excitons in monolayer WSe_2_. Phys. Rev. B. 98, 115308 (2018).

[27] J. van de Groep, J.-H. Song, U. Celano, Q. Li, P. G. Kik, M. L. Brongersma, Exciton resonance tuning of an atomically thin lens. Nat. Photonics 14, 426–430 (2020).

[28] K. L. Seyler, J. R. Schaibley, P. Gong, P. Rivera, A. M. Jones, S. Wu, J. Yan, D. G. Mandrus, W. Yao, X. Xu, Electrical control of second-harmonic generation in a WSe_2_ monolayer transistor. Nat. Nanotechnol. 10, 407–411 (2015). 25895004 10.1038/nnano.2015.73

[29] H.-L. Liu, T. Yang, J.-H. Chen, H.-W. Chen, H. Gao, R. Saito, M.-Y. Li, L.-J. Li, Temperature-dependent optical constants of monolayer MoS_2_, MoSe_2_, WS_2_, and WSe_2_: Spectroscopic ellipsometry and first-principles calculations. Sci. Rep. 10, 15282 (2020). 32943656 10.1038/s41598-020-71808-y PMC7498615

[30] T. Santiago-Cruz, S. D. Gennaro, O. Mitrofanov, S. Addamane, J. Reno, I. Brener, M. V. Chekhova, Resonant metasurfaces for generating complex quantum states. Science 377, 991–995 (2022). 36007052 10.1126/science.abq8684

[31] G. Wang, X. Marie, I. Gerber, T. Amand, D. Lagarde, L. Bouet, M. Vidal, A. Balocchi, B. Urbaszek, Giant enhancement of the optical second-harmonic emission of WSe_2_ monolayers by laser excitation at exciton resonances. Phys. Rev. Lett. 114, 097403 (2015). 25793850 10.1103/PhysRevLett.114.097403

[32] K.-Q. Lin, S. Bange, J. M. Lupton, Quantum interference in second-harmonic generation from monolayer WSe_2_. Nat. Phys. 15, 242–246 (2019).

[33] H. Zhou, M. Qin, H. Xu, G. Wei, H. Li, W. Gao, J. Liu, F. Wu, Photonic spin-controlled self-hybridized exciton-polaritons inWS_2_ metasurfaces driven by chiral quasibound states in the continuum. Phys. Rev. B 109, 125201 (2024).

[34] L. Sortino, A. Gale, L. Kühner, C. Li, J. Biechteler, F. J. Wendisch, M. Kianinia, H. Ren, M. Toth, S. A. Maier, I. Aharonovich, A. Tittl, Optically addressable spin defects coupled to bound states in the continuum metasurfaces. Nat. Commun. 15, 2008 (2024). 38443418 10.1038/s41467-024-46272-1 PMC10914779

[35] R. Verre, D. G. Baranov, B. Munkhbat, J. Cuadra, M. Käll, T. Shegai, Transition metal dichalcogenide nanodisks as high-index dielectric Mie nanoresonators. Nat. Nanotechnol. 14, 679–683 (2019). 31061517 10.1038/s41565-019-0442-x

[36] A. Arora, T. Deilmann, P. Marauhn, M. Drüppel, R. Schneider, M. R. Molas, D. Vaclavkova, S. M. de Vasconocellos, M. Rohlfing, M. Potemski, R. Bratschitsch, Valley-contrasting optics of interlayer excitons in Mo- and W-based bulk transition metal dichalcogenides. Nanoscale 10, 15571–15577 (2018). 30090905 10.1039/c8nr03764g

[37] X. Xu, W. Yao, D. Xiao, T. F. Heinz, Spin and pseudospins in layered transition metal dichalcogenides. Nat. Phys. 10, 343–350 (2014).

[38] N. Bernhardt, K. Koshelev, S. J. U. White, K. W. C. Meng, J. E. Fröch, S. Kim, T. T. Tran, D.-Y. Choi, Y. Kivshar, A. S. Solntsev, Quasi-BIC resonant enhancement of second-harmonic generation in WS_2_ monolayers. Nano Lett. 20, 5309–5314 (2020). 32530635 10.1021/acs.nanolett.0c01603

[39] M. Nauman, J. Yan, D. de Ceglia, M. Rahmani, K. Z. Kamali, C. De Angelis, A. E. Miroshnichenko, Y. Lu, D. Neshev, Tunable unidirectional nonlinear emission from transition-metal-dichalcogenide metasurfaces. Nat. Commun. 12, 5597 (2021). 34552076 10.1038/s41467-021-25717-x PMC8458373

[40] Z. Liu, Y. Xu, Y. Lin, J. Xiang, T. Feng, Q. Cao, J. Li, S. Lan, J. Liu, High-*Q* quasibound states in the continuum for nonlinear metasurfaces. Phys. Rev. Lett. 123, 253901 (2019). 31922806 10.1103/PhysRevLett.123.253901

[41] T. Feng, Y. Xu, W. Zhang, A. E. Miroshnichenko, Ideal magnetic dipole scattering. Phys. Rev. Lett. 118, 173901 (2017). 28498692 10.1103/PhysRevLett.118.173901

[42] A. P. Anthur, H. Zhang, R. Paniagua-Dominguez, D. A. Kalashnikov, S. T. Ha, T. W. W. Maß, A. I. Kuznetsov, L. Krivitsky, Continuous wave second harmonic generation enabled by quasi-bound-states in the continuum on gallium phosphide metasurfaces. Nano Lett. 20, 8745–8751 (2020). 33206536 10.1021/acs.nanolett.0c03601

[43] E. Mobini, R. Alaee, R. W. Boyd, K. Dolgaleva, Giant asymmetric second-harmonic generation in bianisotropic metasurfaces based on bound states in the continuum. ACS Photonics 8, 3234–3240 (2021).

[44] M. R. Shcherbakov, D. N. Neshev, B. Hopkins, A. S. Shorokhov, I. Staude, E. V. Melik-Gaykazyan, M. Decker, A. A. Ezhov, A. E. Miroshnichenko, I. Brener, A. A. Fedyanin, Y. S. Kivshar, Enhanced third-harmonic generation in silicon nanoparticles driven by magnetic response. Nano Lett. 14, 6488–6492 (2014). 25322350 10.1021/nl503029j

[45] G. A. Ermolaev, D. V. Grudinin, Y. V. Stebunov, K. V. Voronin, V. G. Kravets, J. Duan, A. B. Mazitov, G. I. Tselikov, A. Bylinkin, D. I. Yakobovsky, S. M. Novikov, D. G. Baranov, A. Y. Nikitin, I. A. Kruglov, T. Shegai, P. A. Gonzalez, A. N. Gregorenko, A. V. Arsenin, K. S. Novoselov, V. S. Volkov, Giant optical anisotropy in transition metal dichalcogenides for next-generation photonics. Nat. Commun. 12, 854 (2021). 33558559 10.1038/s41467-021-21139-x PMC7870936

[46] H. Zhang, B. Abhiraman, Q. Zhang, J. Miao, K. Jo, S. Roccasecca, M. W. Knight, A. R. Davoyan, D. Jariwala, Hybrid exciton-plasmon-polaritons in van der Waals semiconductor gratings. Nat. Commun. 11, 3552 (2020). 32669550 10.1038/s41467-020-17313-2 PMC7363824

[47] Y. Zhu, B. Wang, Z. Li, J. Zhang, Y. Tang, J. F. Torres, W. Lipiński, L. Fu, Y. Lu, A high-efficiency wavelength-tunable monolayer led with hybrid continuous-pulsed injection. Adv. Mater. 33, 2101375 (2020). 10.1002/adma.202101375 34096112

[48] F. J. F. Löchner, A. George, K. Koshelev, T. Bucher, E. Najafidehaghani, A. Fedotova, D.-Y. Choi, T. Pertsch, I. Staude, Y. Kivshar, A. Turchanin, F. Setzpfandt, Hybrid dielectric metasurfaces for enhancing second-harmonic generation in chemical vapor deposition grown MoS_2_ monolayers. ACS Photonics 8, 218–227 (2021).

[49] P. Hong, L. Xu, M. Rahmani, Dual bound states in the continuum enhanced second harmonic generation with transition metal dichalcogenides monolayer. Opto-Electron. Adv. 5, 200097 (2022).

[50] B. Munkhbat, D. G. Baranov, M. Stührenberg, M. Wersäll, A. Bisht, T. Shegai, Self-hybridized exciton-polaritons in multilayers of transition metal dichalcogenides for efficient light absorption. ACS Photonics 6, 139–147 (2019).

[51] E. Maggiolini, L. Polimeno, F. Todisco, A. D. Renzo, B. Han, M. D. Giorgi, V. Ardizzone, R. Mastria, A. Cannavale, M. Pugliese, V. Maiorano, G. Gigli, D. Gerace, D. Sanvitto, D. Ballarini, Strongly enhanced light–matter coupling of monolayer WS_2_ from a bound state in the continuum. Nat. Mater. 22, 964–969 (2023). 37217703 10.1038/s41563-023-01562-9

[52] T. D. Green, D. G. Baranov, B. Munkhbat, R. Verre, T. Shegai, M. Käll, Optical material anisotropy in high-index transition metal dichalcogenide Mie nanoresonators. Optica 7, 680–686 (2020).

[53] Z. Zheng, D. Rocco, H. Ren, O. Sergaeva, Y. Zhang, K. B. Whaley, C. Ying, D. de Ceglia, C. De-Angelis, M. Rahmani, L. Xu, Advances in nonlinear metasurfaces for imaging, quantum, and sensing applications. Nanophotonics 12, 4255–4281 (2023). 39634716 10.1515/nanoph-2023-0526 PMC11501303

[54] Y. Kivshar, All-dielectric meta-optics and non-linear nanophotonics. Natl. Sci. Rev. 5, 144–158 (2018).

[55] M. A. Weissflog, A. Fedotova, Y. Tang, E. A. Santos, B. Laudert, S. Shinde, F. Abtahi, M. Afsharina, I. P. Perez, S. Ritter, H. Qin, J. Janousek, S. Shradha, I. Staude, S. Saravi, T. Pertsch, F. Setzpfandt, Y. Lu, F. Eilenberger, A tunable transition metal dichalcogenide entangled photon-pair source. Nat. Commun. 15, 7600 (2024). 39217175 10.1038/s41467-024-51843-3 PMC11366010

[56] G. Zograf, A. Y. Polyakov, M. Bancerek, T. J. Antosiewicz, B. Küçüköz, T. O. Shegai, Combining ultrahigh index with exceptional nonlinearity in resonant transition metal dichalcogenide nanodisks. Nat. Photonics 18, 751–757 (2024).

[57] G. Zograf, B. Küçüköz, Alexander Yu. Polyakov, M. Bancerek, A. V. Agrawal, W. Wieczorek, T. J. Antosiewicz, T. O. Shegai, Ultrathin 3R-MoS_2_ metasurfaces with atomically precise edges for efficient nonlinear nanophotonics. arXiv:2410.20960 [physics.optics] (2024).

[58] Z. Han, F. Ding, Y. Cai, U. Levy, Significantly enhanced second-harmonic generations with all-dielectric antenna array working in the quasi-bound states in the continuum and excited by linearly polarized plane waves. Nanophotonics 10, 1189–1196 (2021).

[59] M. Kjellberg, F. Vennberg, A. P. Ravishankar, S. Anand, Polarization-enabled tuning of anapole resonances in vertically stacked elliptical silicon nanodisks. Adv. Photonics Res. 5, 2400009 (2024).

[60] Z. Huang, J. Wang, W. Jia, S. Zhang, C. Zhou, High-*Q* all-dielectric metasurface perfect absorber powered by quasi-bound states in the continuum. Appl. Phys. Lett. 125, 141702 (2024).

[61] Y. Li, A. Chernikov, X. Zhang, A. Rigosi, H. M. Hill, A. M. van der Zande, D. A. Chenet, E.-M. Shih, J. Hone, T. F. Heinz, Measurement of the optical dielectric function of monolayer transition-metal dichalcogenides: MoS_2_, MoSe_2_, WS_2_ and WSe_2_. Phys. Rev. B 90, 205422 (2014).

[62] T. J. Kim, V. L. Le, H. T. Nguyen, X. A. Nguyen, Y. D. Kim, Modeling of the optical properties of monolayer WS_2_. J. Korean Phys. Soc. 77, 298–302 (2020).

