# Supplementary material for: Dynamic control of nonlinear emission by exciton-photon coupling in WS2 metasurfaces
Source: Sci Adv. 2025 Aug 29;11(35):eady2108. doi: 10.1126/sciadv.ady2108 (PMC12396309; doi:10.1126/sciadv.ady2108)
Supplement: Supplementary file 1 — Figs. S1 to S15 Supplementary Text References [file sciadv.ady2108_sm.pdf]

Science Advances
AAAS

Supplementary Materials for

Dynamic control of nonlinear emission by exciton-photon coupling in WS_2 metasurfaces

Mudassar Nauman *et al.*

Corresponding author: Dragomir Neshev, dragomir.neshev@anu.edu.au; Yuerui Lu, yuerui.lu@anu.edu.au

Sci. Adv. **11**, eady2108 (2025)
DOI: 10.1126/sciadv.ady2108

This PDF file includes:

Figs. S1 to S15
Supplementary Text
References

Metasurface Fabrication

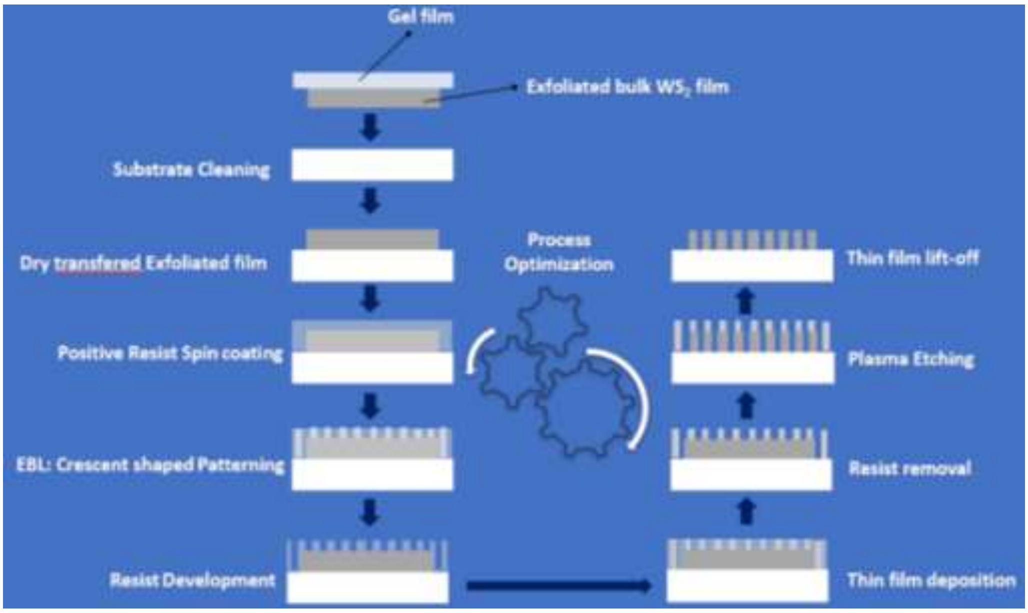

Fig. S1. Schematic illustration of the fabrication process. For crescent-shaped WS_2 metaatoms on a sapphire substrate, including steps such as exfoliation of bulk WS_2, substrate cleaning, dry transfer, electron beam lithography patterning, metal deposition, etching, and lift-off to create the final metaatom arrays.

The fabrication of crescent-shaped WS_2 metaatoms on a sapphire substrate was carried out using electron beam lithography (EBL) in the cleanroom facilities at the Australian National Fabrication Facility Labs. The schematic of complete fabrication process and the steps involved in the process are shown in fig. S1 and discussed as follows:

First, we exfoliated multiple bulk WS_2 films from HQ-Graphene, 2H-WS_2 crystals on gel-films (Gel-Pak) and then exfoliated bulk WS_2 films were dry transferred onto sapphire substrates. Then we measured the thickness of each film using a Dektak Surface Profilometer. Next, we chose a film with a uniform thickness of approximately 220 nm over an area exceeding 400 µm × 400 µm for our qBIC metasurface design. Note: prior to transferring the exfoliated bulk WS_2 film to the sapphire substrate, the substrate was cleaned by sonicating in acetone for 3 minutes, followed by isopropanol (IPA) for 3 minutes, and deionized (DI) water for 3 minutes. The substrate was then dried with nitrogen gas and subjected to 30 seconds of O_2 plasma cleaning in a barrel etcher to remove any surface contamination. The exfoliated WS_2 film was then dry transferred onto the sapphire substrate using a home-built dry transfer setup.

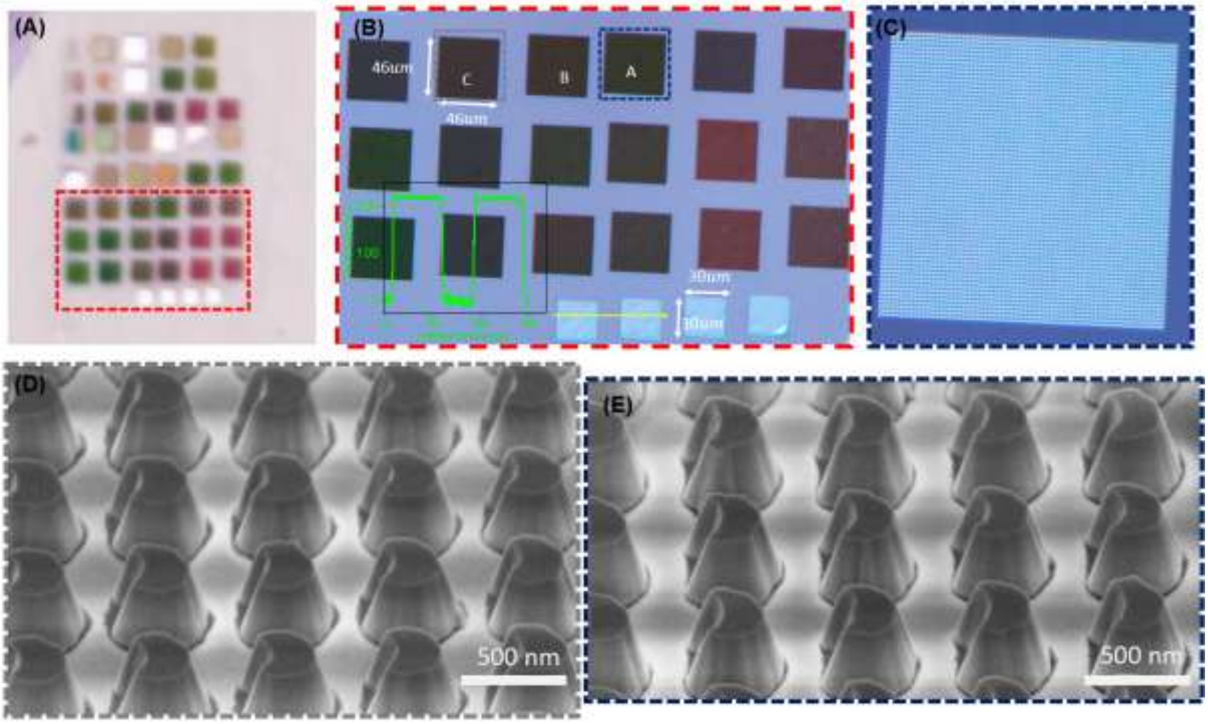

Fig. S2. Fabricated WS_2 metasurfaces with unpatterned reference regions for SHG enhancement calibration: (**A**) Patterned bulk WS_2 flake on sapphire substrate. (**B**) Showing patterned metasurfaces A, B, C, and unpatterned WS_2 films. The array size of the metasurfaces is $46um \times 46um$. Intentionally unpatterned WS_2 films of size $30um \times 30um$ are visible at the bottom of the image. The green arrow marks the lateral scan path for Dektak profilometry. *Inset:* Measured thickness (220 nm) of two unpatterned films, confirming uniformity. (**C**) Optical image of entire metasurface A. (D) SEM image of metasurface C. (E) SEM image of the metasurface A. The SEM and optical images illustrate the high quality fabrication of the metasurfaces, highlighting colour uniformity and precise nanostructuring.

After film transfer, the sample was cleaned by sequential rinsing in acetone, IPA, and DI water, each for 30 seconds, to remove any residues. A single layer of positive resist (ZEP 520A) was then spin-coated onto the sample. Crescent-shaped metaatom arrays were patterned via EBL (20 kV acceleration voltage, 10 μm aperture size), followed by development in ZEP developer to remove the exposed areas, thereby creating the crescent-shaped metaatom arrays. Next, a 50 nm thick layer of aluminum was deposited onto the sample using e-beam evaporation. The sample was then immersed in ZEP remover to lift off the resist and metal film, leaving the arrays of crescent-shaped metaatoms (each measuring approximately 46 μm × 46 μm) formed from the aluminum deposited directly onto the WS_2 film.

In addition to the crescent metaatoms, square patches (approximately 30 μm × 30 μm) were patterned on the same film to serve as unpatterned reference regions. Dry etching was performed using an inductively coupled plasma (ICP) tool with a plasma of Ar, CHF_3, and SF_6 gases. The etching rate of the WS_2 film was optimized to 60 nm/min, and a total etching time of 3 minutes and 36 seconds was used, with an additional 10% over-etch to ensure complete pattern transfer. The etching process removed the WS_2 film everywhere except under the aluminum masks (arrays of crescent-shaped

metaatoms and square patches). Finally, the remaining aluminum mask was removed by etching in an aluminum etchant.

Each step of the fabrication process was carefully optimized to achieve the desired patterning and high-quality metaatom arrays and unpatterned WS_2 films to serves as reference regions, as illustrated in fig. S2.

Linear Response

Refractive Index of Bulk WS_2

To design a WS_2 metasurface, we first need to determine the refractive index of bulk WS_2. To obtain this, we exfoliated a 220 nm thick WS_2 film onto a sapphire substrate and measured its transmission response using our transmission spectroscopy technique. We then applied a fitting procedure to extract the refractive index values, as shown in fig. S3.

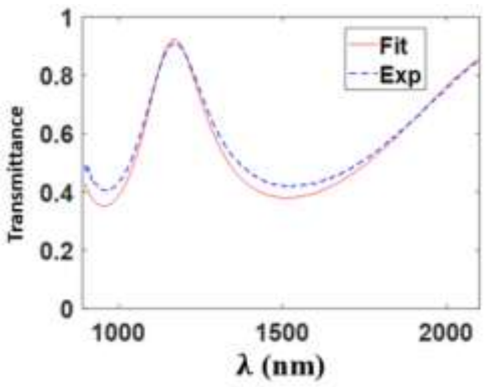

Fig. S3. Measured linear response of 220 nm thick WS_2 film. Transmission spectra of a 220 nm thick WS_2 film on sapphire substrate, with results used to determine the refractive index values.

Transmission and Multipole Decomposition

In fig S4(A), we illustrate the design strategy for the crescent metaatom. The spectral position of the qBIC resonance can be tuned by adjusting the asymmetry of the metaatom (58). This asymmetry can be modified either by changing δ or δ''. In our designs, we controlled the asymmetry by varying δ'' while keeping δ constant. For instance, metasurface A has δ = 170 nm and δ'' = 200 nm, whereas metasurfaces B and C were obtained by altering δ'' alone. We have done the multipole decomposition simulations in COMSOL Multiphysics, to break down the electromagnetic response of our metasurface into its multipolar components such as electric dipole (ED), magnetic dipole (MD), and higher order modes like electric quadrupole (EQ), and magnetic quadrupole (MQ), as depicted in fig. S4A. The multipole decomposition clearly shows the WS_2 crescent metaatom acts as a pure MD. Typically, high-refractive-index subwavelength nanoparticles exhibit both electric and magnetic dipole responses. To achieve pure MD scattering, it is crucial to suppress the ED response at the same spectral position. This can be accomplished through the anapole mode (59), which is characterized by the absence of ED

scattering. By spectrally aligning the magnetic dipole resonance with the anapole mode, we can achieve ideal MD type strong scattering in the far field, exhibiting MD type qBIC resonance in the near-infrared spectrum, as demonstrated in fig. S4B and S4C. We applied Fano-fitting to calculate the the Q-factor of the measured qBIC resonance which is 43.6 and simulated Q-factor is 110, as illustrated in fig. S4C. The difference in the simulated and measured Q-factor is due to the difference in surface roughness of the simulated and fabricated metaatoms.

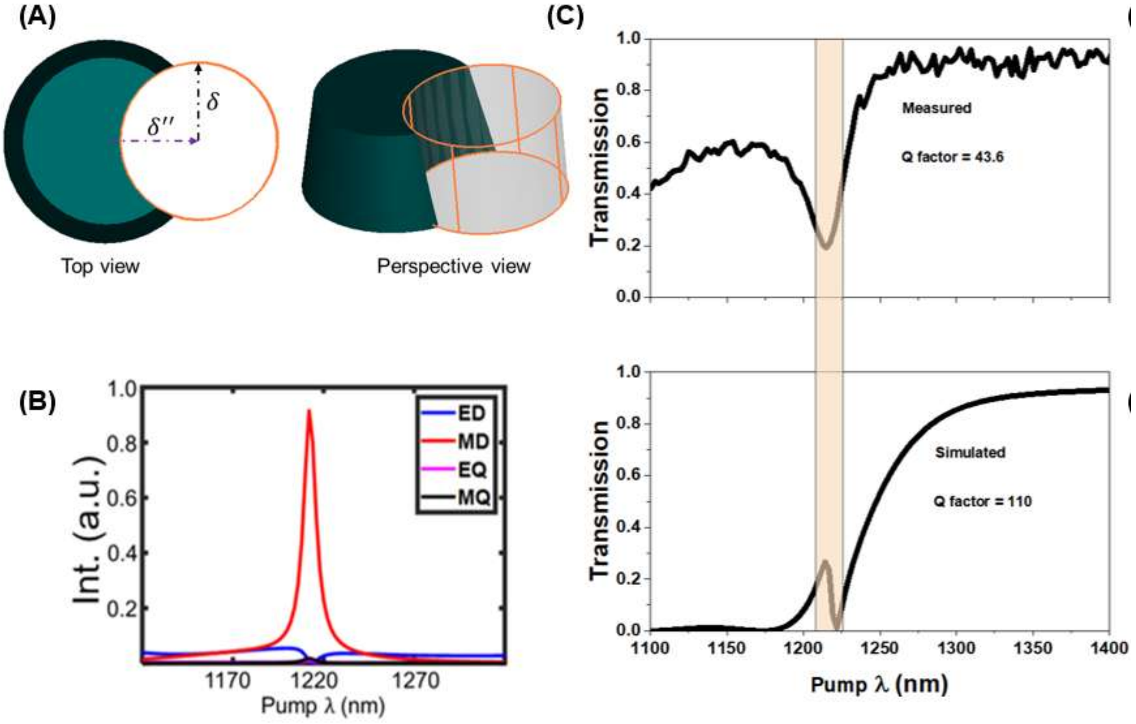

Fig. S4. Linear response of metasurface A. (**A**) Top and perspective view of designed crescent metaatom: δ represents the radius of the cylindrical cavity and δ'' is displacement parameter. (**B**) Multipole decomposition of metasurface A. (**C**) Comparison between measured and simulated normalized transmission intensity.

qBIC Excitation as a Function of Incident Light Polarisation.

Intriguingly, the MD type qBIC excitation can be tuned all optically at the same spectral position, as function of incident light polarization angle, φ, as depicted in fig. S5A. This tunability in our metasurface is due to the asymmetric coupling of induced qBIC mode with incident light (60). A strong resonance with high Q-factor is excited in transmission spectrum, as depicted in fig. S5B, when the light's polarisation is aligned with one of the main axes (Y-axis) of crescent shaped metaatoms, as shown in fig. S5A. If the polarisation of incident light is aligned with another axis (X-axis), as shown

in fig. S5A, the interaction is weaker, resulting in resonance disappearing, as shown in fig. S5B. So, the asymmetric coupling is the variation in the coupling strength depending on the direction. The resonance intensity can be adjusted by changing the polarisation angle without shifting its spectral position.

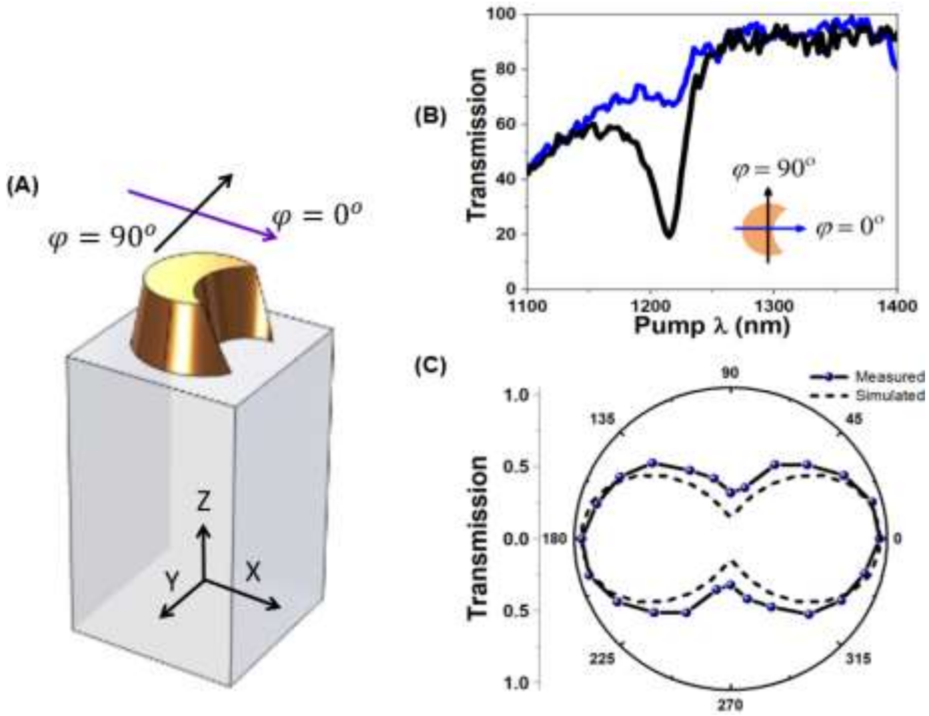

Fig. S5. Tunable qBIC as function of pump polarisation. (A) Schematic showing excitation of qBIC as function of incident light polarization angle Y-axis ($\varphi = 90^\circ$), and X-axis ($\varphi = 0^\circ$). (B) When $\varphi = 90^\circ$, a dip in transmission spectrum at 1220 nm indicates qBIC excitation , and when $\varphi = 0^\circ$ then no dip in transmission spectrum showing qBIC switched off, the inset showing the polarization angle of incident light on our metasurface unit cell. (C) Polar graph of measured and simulated normalized transmission intensity as function of polarization angle from 0° to 360°: minima in transmission spectra on polar graph indicates qBIC excitation at $\varphi = 90^\circ$ and maxima indicates qBIC switched off at $\varphi = 0^\circ$.

For further verification, we measured and simulated transmission spectra of the metasurface A as function of incident light polarisation angle (φ) with step size of $15^\circ$and plotted data on polar axis, as shown in the fig. S5C. It can be observed in fig. S5C that the resonance intensity of qBIC resonance can be varied (exhibiting coupling strength with the incident light) as a function of φ. We can trigger a strong pure MD type qBIC resonance on and off all optically by just controlling φ. We found a very good agreement between experimental and simulated results, as shown in fig. S5C.

Despite the absence of a qBIC in the transmission spectrum, the SHG enhancement for x-polarized light remains higher than that of the unpatterned WS_2 film. This can be attributed to several factors, including localized field enhancements or weak resonances within the metasurface. Additionally, the metasurface

may couple to higher-order or off-resonant modes, which still contribute to the SHG process, albeit with lower efficiency compared to the qBIC-driven mechanism. These effects collectively enable significant SHG enhancement even without a dominant qBIC resonance.

Nonlinear Response

Wavelength dependent SHG

To investigate the influence of resonant conditions between the qBIC and the exciton A (E_o^A) in WS_2 metasurfaces, we performed wavelength-dependent SHG measurements on metasurfaces A, B, and C, as shown in fig. S6. We employed a tunable femtosecond laser (Chameleon Ultra II and OPO, pulse width of ~200 fs) in a home-build microscopy setup. We performed wavelength-dependent nonlinear measurements by focusing the laser beam onto the metasurface using a 5× microscope objective (NA = 0.2). We then collect the SH in the forward direction by a second objective (20×, NA = 0.4). To filter out the transmitted and reflected pump wave, we employed a short-pass filter at 800 nm.

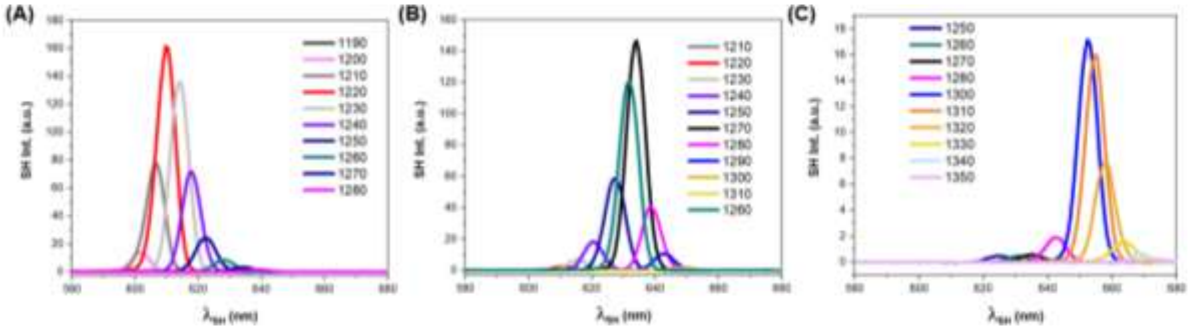

Fig. S6. Wavelength dependent SHG of metasurfaces at room temperature. (**A**) Metasurface A. (**B**) Metasurface B. (**C**) Metasurface C.

Two waveplates are used to control the polarisation of the pump beam. The wavelength of the infrared pump beam was scanned over the range of 1190-1350 nm. The measured SHG response for each pump wavelength is depicted in fig. S6. Metasurfaces A, B, and C exhibit qBICs at 1220 nm, 1270 nm, and 1305 nm, respectively. The SHG response is enhanced when the pump wavelength resonates with the qBIC positions of the metasurfaces. However, a significantly greater enhancement in the SHG response is observed when a metasurface (metasurface A) meets the double resonant condition $\lambda_{qBIC} = 2 \times \lambda_{E_o^A}$, as illustrated in fig. S6A.

Power-dependent SHG

We performed power-dependent SHG measurements on metasurface A, to validate the second order nonlinear response and its efficiency under varying pump power. The measured power dependent SHG on metasurface A shows a clear quadratic relationship between the SHG signals and pump power, as illustrated in fig. S7. This behaviour exhibits the second order nonlinear nature of SHG, where the intensity of SH scales with square of the pump power, as shown in fig. S7. Interestingly, no saturation

of the emitted SH signals observed in fig. S7 when the pump average power is increased up to 100 mW. This shows that the proposed metasurface maintains consistent nonlinear response even at higher power levels, confirming efficient SHG without degradation or loss of performance.

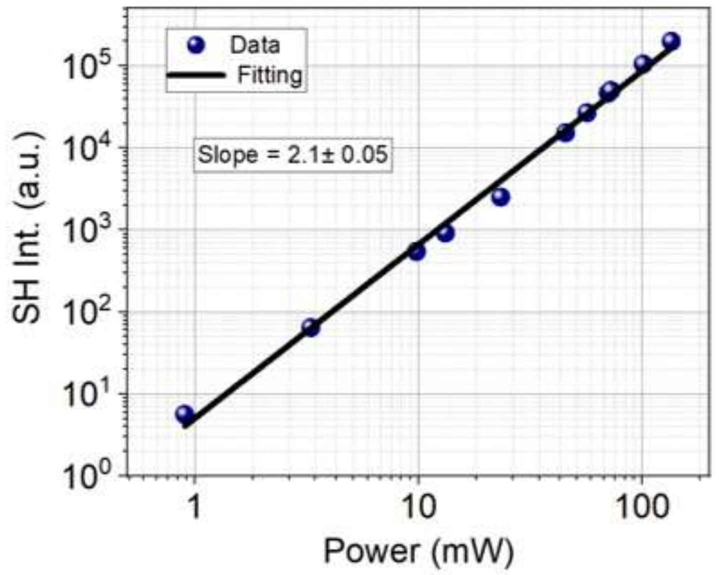

Fig. S7. Power dependent second harmonic generation.

SHG efficiency comparison of metasurface A with WS_2 monolayer

We exfoliated WS_2 monolayer (1L-WS_2) from the same bulk crystals (HQ Graphene 2H-WS_2, which were used in our metasurface study) and transferred it onto a sapphire substrate, as shown in fig. S8A, using a home-built dry transfer setup. The photoluminescence (PL) image of the exfoliated 1L-WS_2 was captured using a 532 nm CW-laser in a WiTEC confocal laser PL microscopy system, which revealed a strong and uniform PL signal at ~610 nm due to the direct bandgap nature of the material, as shown in fig. S8.

To investigate the second-harmonic generation (SHG) properties of the monolayer, in our experiments, we used the same optical bench system for both the WS_2 metasurface and monolayer measurements, but with different objectives tailored to each setup. For the metasurface, we employed a 5x objective (NA: 0.14) for excitation, providing a larger field of view and broader illumination area for uniform excitation. A 20x objective (NA: 0.4) was used for collection, enhancing SHG signal capture and spatial resolution. In contrast, for the WS_2 monolayer, we used a 100x objective (NA: 0.7) for both excitation and collection. This higher magnification and NA were necessary to focus the excitation light into a smaller spot size, increasing local intensity and boosting the SHG efficiency, which is crucial for the thin monolayer material. Despite this difference, all other experimental setups were the same.

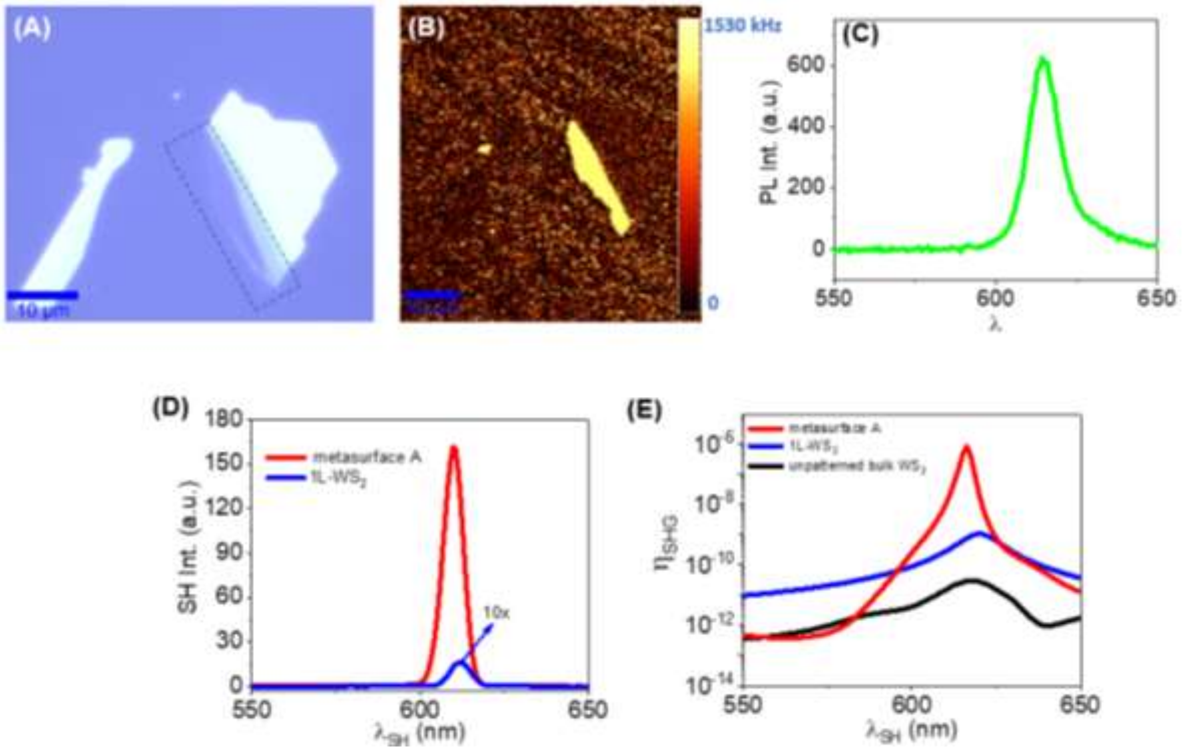

Fig. S8. SHG efficiency comparison of metasurface A and 1L-WS$_2$. (**A**) Optical image of 1L-WS$_2$. (**B**) PL mapping. (**C**) PL spectra, showing peak intensity around 613 nm. (**D**) Measured SH intensity comparison of metasurface A (with qBIC at 1220 nm) and 1L-WS$_2$, the metasurface A SH intensity is 98-folds stronger than 1L-WS$_2$. (**E**) Simulated SHG efficiency (η_{SHG}) comparison of metasurface A, 1L-WS$_2$, and unpatterned bulk WS$_2$ film.

A comparison of SHG intensity between metasurface A and 1L-WS$_2$ is shown in fig. S8D. Notably, the SHG signal from the qBIC-enhanced WS$_2$ metasurface A was found to be more than 98-fold stronger than that from the 1L-WS$_2$. To further corroborate our experimental results, we performed full-wave numerical simulations in COMSOL and excited the metasurface A, 1L-WS$_2$, and unpatterned bulk WS$_2$ film with peak intensity of 1 GWcm^{-2}, which indicated that the SHG efficiency of the metasurface A is 3 orders of magnitude stronger than that from the monolayer and 5 orders of magnitude stronger than unpatterned bulk WS$_2$ film, as shown in fig. S8E. The discrepancy between experimental and simulated results can be attributed to following factors: the differences in measured and simulated qBIC Q-factors and excitation conditions as well. The SHG intensity (I_{SHG}) scales quadratically with the fundamental pump intensity (I_{Pump}). The measured local pump intensity for the monolayer case is 11 times higher than the metasurface's measured local intensity of 0.93 $GWcm^{-2}$. Since the SH intensity (I_{SH}) is proportional to the square of local pump intensity (I_{pump}) as $I_{SH} \propto \left(I_{pump}\right)^2$. Therefore, if we illuminate our metasurface with the same local pump intensity used to measure SHG from the 1L-WS$_2$,

the observed SHG enhancement of the metasurface relative to 1L-WS_2 will align more closely with the simulated values.

Polarization resolved SHG

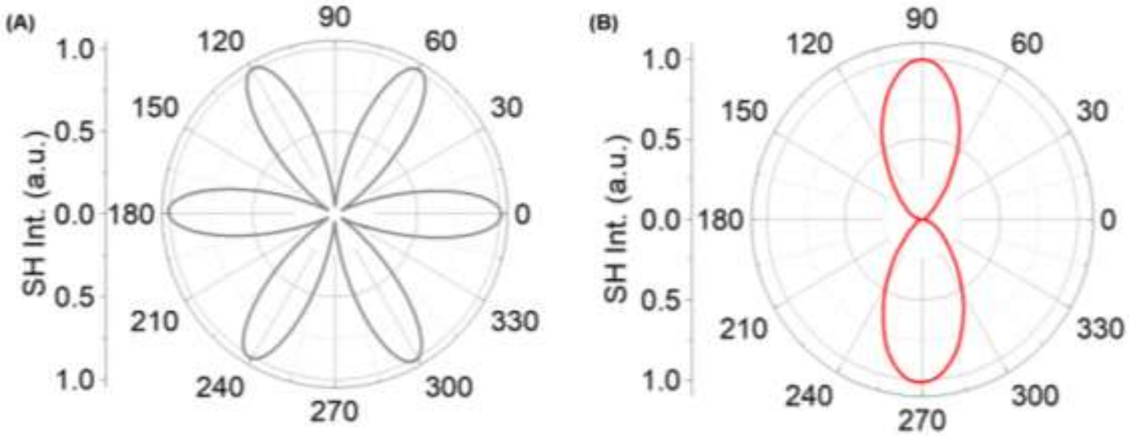

Fig. S9. Measured polarisation resolved SHG: (**A**) unpatterned bulk WS_2 film. (**B**) Metasurface A.

To measure the polarisation resolved SHG, we excited unpattern bulk WS_2 film with pump wavelength of 1220 nm and then changed pump polarisation angle (φ) from 0° to 360°. It can be observed due to hexagonal orientation of lattice atoms in bulk WS_2, the polarisation resolved SHG follows symmetric six-fold SHG radiation pattern, as shown in fig. S9A. The measured polarisation resolved SHG as function of φ of metasurface A (at pump wavelength of 1220 nm) is depicted in fig. S9B. The metasurface A modulate the six-fold rotational pattern of unpatterned WS_2 film into dipole like SHG pattern because the induced qBIC sensitive to the φ of the incident light.

Nonlinear Modelling.

We further performed full-wave numerical simulations to assess the tunable enhanced SHG from metasurface A (enabled by double resonant condition between qBIC and E_o^A). In numerical simulations we will show that the double resonant condition ($\lambda_{qBIC} = 2 \times \lambda_{E_o^A}$) can be restored and broken either by spectral tuning/detuning of qBIC via physical change in the structure or by spectral tuning/detuning of E_o^A via an external stimulus. By doing this we will be able to confirm our proof of concept that our unique approach of doubly resonant condition has not only helped us to significantly enhance the SHG but also to make this enhancement tunable, which is crucial for next-gen reconfigurable nonlinear meta-optics. We have adopted two approaches, in the first approach, in our simulations we will change the design to spectrally tune the qBIC at pump wavelengths to break and restore the double resonant condition and then show significant SHG enhancement at RT when qBIC will meet the double resonant condition. Second, the E_o^A can be tuned at SH wavelengths via an external stimulus (temperature in our study), thereby enabling us to break and restore the double resonant condition and significant enhancement in SHG at RT when E_o^A will meet double resonant condition.

In our modelling, near the SH wavelengths, where E_O^A dominates the optical response, we employed model described in (61). This model accounts for all the material resonances to accurately represents the optical behaviour in the visible spectrum.

Spectral tuning of qBIC at pump wavelengths

To detune the qBIC along longer and shorter wavelengths in metasurface A, we can change the asymmetry percentage by just changing the displacement parameter δ'' along one of the xy axis, x-axis in our case, and kept all other parameters constant.

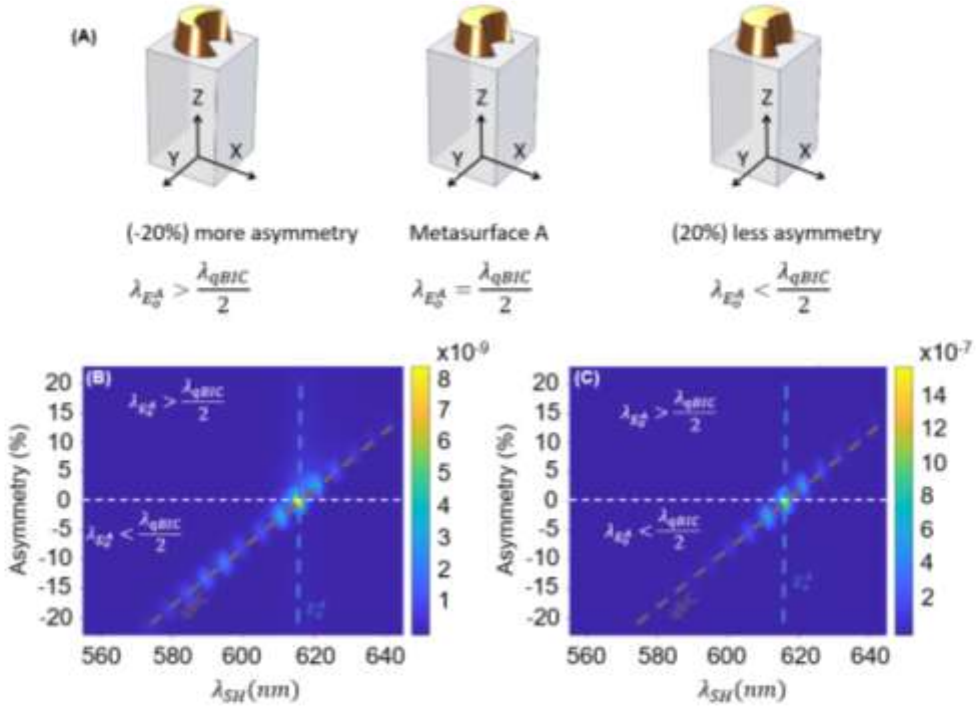

Fig. S10. Calculated SHG directionality. (**A**) Schematic of varying the asymmetry factor percentage of crescent shaped metaatoms with respect to the metasurface A, positive and negative values of asymmetry(%) would represent less and more asymmetry in reference to the metasurface A, respectively. (B) Calculated SHG efficiency in reflection mode. Horizontal black dashed line represent the region where $\lambda_{E_O^A} = \frac{\lambda_{qBIC}}{2}$, meeting virtual interaction condition between E_O^A and qBIC. (C) Calculated SHG efficiency in transmission mode.

The fig. S10A illustrating the schematic of metasurfaces with 20% less asymmetry (right in the panel), and 20% more asymmetry (left in the panel) in comparison to the metasurface A (middle in the panel). In comparison to the metasurface A (where $\lambda_{E_O^A} = \frac{\lambda_{qBIC}}{2}$: a condition for virtual interaction), 20% more asymmetry will break virtual interaction condition by tuning qBIC resonance towards the shorter wavelengths (where $\lambda_{E_O^A} > \frac{\lambda_{qBIC}}{2}$). Similarly, 20% less asymmetry would also break virtual interaction condition by moving qBIC resonance along the longer wavelength (where $\lambda_{E_O^A} < \frac{\lambda_{qBIC}}{2}$). If we say the

δ'' for the cone of our metasurface A as δ''(A) and for the new cone as δ''(N) then we can use following simple formula to calculate the asymmetry percentage with reference to the metasurface A.

$$Asymmetry\ (\%) = \left(\frac{x\ position\ of\ \delta''(A) - x\ position\ of\ \delta''(N)}{x\ position\ of\ \delta''(A)}\right) \times 100.$$

We calculated SHG efficiency as a function of asymmetry percentage and excitation wavelength in reflection mode fig. S10B and in transmission mode fig. S10C. It can be observed that SHG enhancement occurs at the q-BIC resonance, but the maximum peak occurs when $\lambda_{E_O^A} = \frac{\lambda_{qBIC}}{2}$, as shown in fig. S10B and fig. S10C. Interestingly, SHG in the transmission is much stronger (2 orders of magnitude) than SHG in reflection, as shown in fig. S10B and fig. S10C.

Spectral tuning of exciton at SH wavelengths

For spectral tuning of E_O^A at SH wavelengths, we applied temperature as an external stimulus in our study. In our nonlinear model, the temperature dependence of the WS_2 permittivity was taken from (62) ,where a parametric model using cubic polynomials was introduced to fit spectroscopic ellipsometry data. The wavelength and temperature dependence of the optical properties of WS_2 are reported in fig. S11 near the A exciton wavelength (at approximately 600 nm).

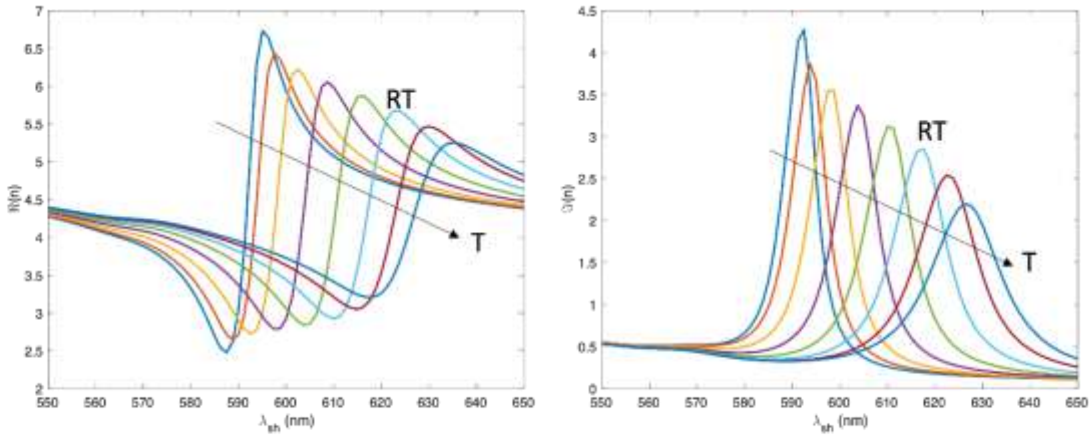

Fig. S11. Wavelength and temperature dependent optical properties of WS_2.

The second-order non-linear susceptibility was retrieved by the linear properties by adopting the Miller's rule, i.e., by assuming that $\chi^{(2)}(2\omega, \omega, \omega) \sim \chi^1(2\omega)[\chi^1(\omega)]^2$. In particular, if one approximates the optical response of WS_2 near 600 nm as a single Lorentz oscillator, corresponding to exciton A, with temperature-dependent resonance frequency (ω_A) and linewidth (γ_A) taken from (62) the nonlinear susceptibility takes the wavelength- and temperature-dependence form reported in fig. S12.

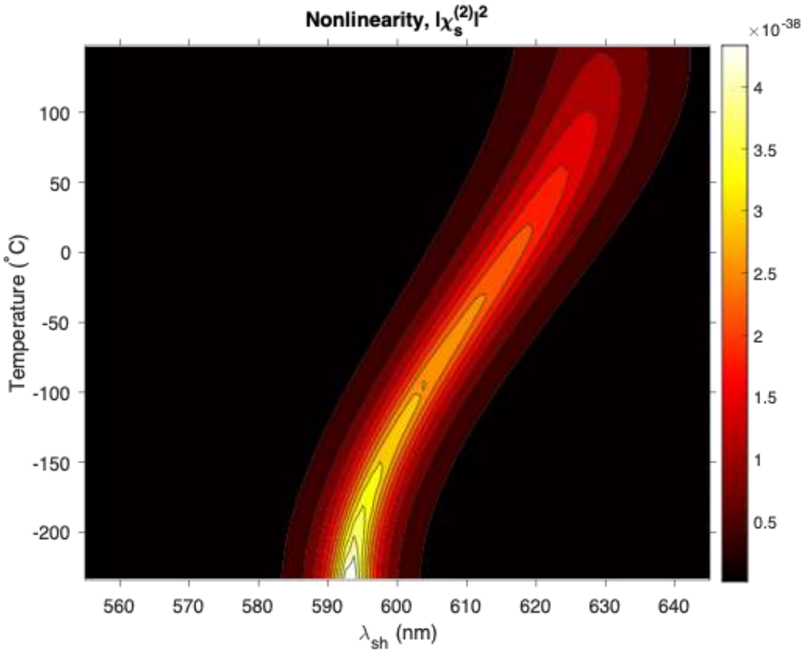

Fig. S12. Wavelength and temperature dependent nonlinear susceptibility of WS_2.

It is important to stress that, while temperature variations induce large changes of linear and nonlinear susceptibility around the A exciton wavelength (near 600 nm), variations of refractive index are negligible near the quasi-BIC resonance in the infrared (i.e., around 1200 nm). In other words, temperature tunability of SHG is mainly associated to the temperature modulation of $\chi^1(2\omega)$. Reflected and transmitted SHG conversion efficiencies were then numerically calculated using the linear and nonlinear optical properties presented in fig. S11 and fig. S12 in a finite-element simulator (COMSOL Multiphysics). One unit cell of the periodic metasurface was simulated with continuity conditions for the fields at the boundaries of the unit cell in the directions of the structure periodicity. At the pump frequency, a normally-incident plane-wave port was set on the air side and a receiving plane-wave port was set on the transmission side in the sapphire substrate region. The solution at the pump-frequency problem was used to calculate the induced second harmonic currents in the meta-atom. In particular, as outlined in (main manuscript: (39)) , the second-harmonic current in the bulk WS_2 was set to zero (since WS_2 is a centrosymmetric material), while the following surface currents were considered at the top and bottom surfaces: $J_{NL}(2\omega) = -i2\omega\epsilon_0\chi^{(2)}\left[\left(E_x^2 - E_y^2\right)\hat{x} - 2E_xE_y\hat{y}\right]$,

Where E_x and E_y are the x and y components of the pump electric field, and $\chi^{(2)}$ is the wavelength- and temperature dependent function described above (illustrated in fig. S12). Perfectly matched boundary conditions are set on the air and sapphire sides of the simulation problem at the second harmonic in order to absorb higher orders of diffraction.

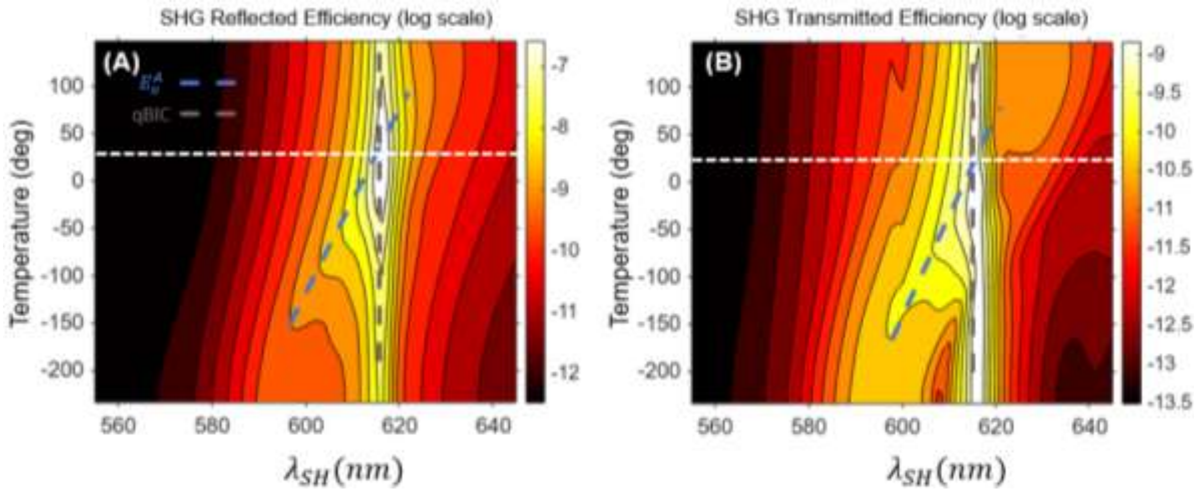

Fig. S13. Simulated wavelength and temperature dependent SHG of metasurface A over a large temperature range of 100 deg to −190 deg. (**A**) SHG efficiency in reflection**.** (**B**) SHG efficiency in transmission. In (**A**) and (**B**), white dashed line is an eye guide for region where E_o^A interacts with $\frac{\lambda_{qBIC}}{2}$ at room temperature (RT ~23 deg), exhibiting E_o^A and qBIC are in double resonant condition ($\lambda_{qBIC} = 2 \times \lambda_{E_o^A}$), thereby enabling significant SHG enhancement

Fig. S13 depicts the simulated SHG of metasurface A in both reflection and transmission. It can be observed in fig. S13 that variations in temperature relative to RT result in a spectral shift in the E_o^A spectral position ($\sim 610\, nm$ at RT), towards longer wavelengths with an increase and shorter wavelengths with a decrease. This spectral shift breaks and subsequently restores the double resonant condition established at RT (represented by white dashed line) between qBIC and E_o^A, leading to significant SHG enhancement. As the E_o^A moves away from its resonance with qBIC, either to longer or shorter wavelengths, the SH intensity starts reducing. The reflected (fig. S13A) and transmitted (fig. S13B) SHG shows a pronounced maxima at $\sim 610\, nm$ at RT where E_o^A virtually interacts with $\frac{\lambda_{qBIC}}{2}$, fulfilling double resonant condition, as illustrated in fig. S13. Moreover, It can also be observed in fig. S13 that the transmitted SHG, fig. S13B, is approximately two orders of magnitude larger than the reflected one, fig. S13A. Variations of temperature can significantly alter the emitted second harmonic light, both in reflection and transmission.

Fermi's golden rule

A better understanding of the doubly resonant condition enhanced SHG and its dependence on qBIC spectral position (as function of asymmetry) and an external stimulus such as temperature can be gained by considering the Femi's golden rule for the SHG conversion efficiency, according to which the efficiency of SHG scales as follows:

$$\eta_{SHG} \propto \left|\chi^{(2)}\right|^2 L^4(\omega) L^2(2\omega) \xi \qquad (1)$$

Where $L(\omega)$ and $L(2\omega)$ are the field enhancement factors at the pump and SH frequencies, respectively, $\chi^{(2)}$ represents inherent material nonlinearity, which in our proposed metasurface is expressed by A exciton (E_O^A), ξ overlap integral between pump and SH fields. In our problem, $L(\omega)$, the field enhancement at the pump frequency (expressed by qBIC at pump frequency), and it has strong dependence on asymmetry instead of temperature (variations of refractive index are negligible near the quasi-BIC resonance in the infrared). In contrast to $L(\omega)$, $\chi^{(2)}$ is highly dependent on temperature and wavelength, as illustrated in fig. S12. If we neglect, for simplicity, the effects of temperature dependence of $L(2\omega)$ and ξ, the simple product of the susceptibility term and the pump field enhancement term in equation (1), is enough to qualitatively explain the measured SHG efficiencies, reported in the main text (Fig. 4) and calculated SHG efficiencies, illustrated in fig. S10. The new modified equation (2) given below can help us explain the enhanced SHG at RT due to doubly resonant condition.

$$\eta_{SHG} \propto \left|\chi^{(2)}\right|^2 L^4(\omega) \tag{2}$$

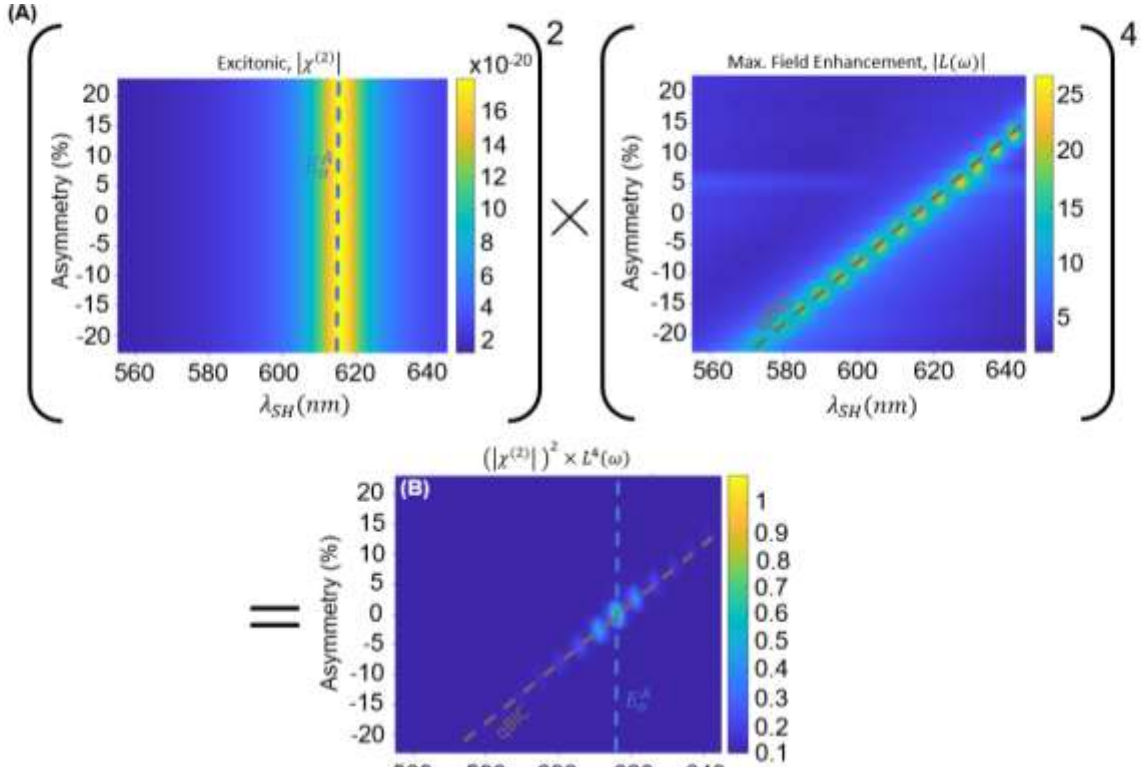

Fig. S14. Calculated SHG efficiency via Fermi's golden rule. (**A**) SHG efficiency obtained by separately considering the effects of $\chi^{(2)}$ and $L(\omega)$. Represents right hand side of equation (2). (**B**) resultant SHG efficiency derived from right hand side of equation (2), represents left hand side of equation (2).

At RT, the SHG efficiency can be separately calculated by considering $\chi^{(2)}$, which is strongly related to E_0^A and independent of asymmetry, versus the asymmetry of the metaatoms, as depicted in the left panel of fig. S14A. Similarly, $L(\omega)$ versus asymmetry is shown in the right panel of fig. S14A. Applying the right-hand side of equation (2) yields the resultant SHG efficiency, as illustrated in fig. S14B, which corresponds to the left-hand side of the equation. This resultant SHG efficiency is consistent with the calculated and measured values shown in fig. S11 and the main manuscript Fig. 4(A), respectively.

For temperature-dependent SHG efficiency calculations, only the $\chi^{(2)}$ term in equation (2) demonstrates significant dependence on wavelength and temperature, as presented in fig. S12, whereas $L(\omega)$, strongly linked to qBIC, remains unaffected, as shown in fig. S13. By taking the simple product of the SHG efficiency term $\left|\chi^{(2)}\right|^2$ (displayed in fig. S12) and $L(\omega)$, the resultant SHG efficiency aligns with the results illustrated in Fig. 4(D) of the main manuscript. This approach sufficiently and qualitatively explains the SHG efficiencies derived from full-wave nonlinear simulations.

Measured SHG Efficiency:

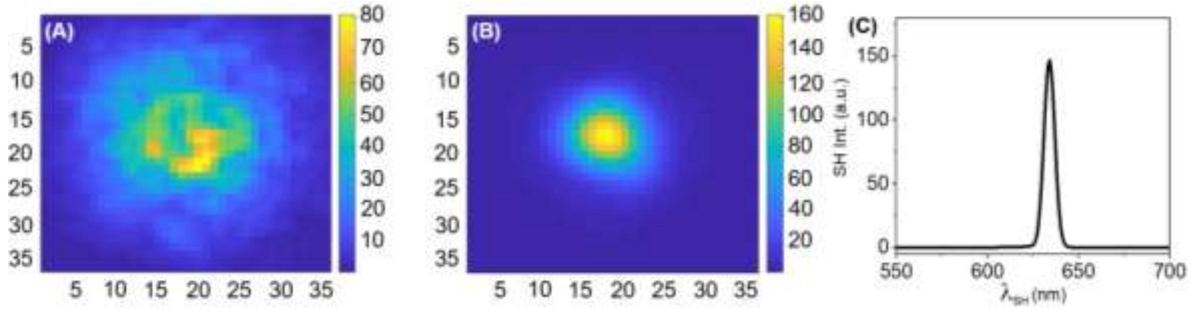

Fig. S15. SHG efficiency estimation. (**A**) SHG image from camera with 10 ms integration. (**B**) Calibration laser at 633 nm image from camera with 10 ms integration time. (**C**) The SHG spectrum of metasurface B by 1270 nm pump laser.

We used Thorlabs CMOS camera with 10 ms integration time to obtain SHG intensity, as shown in fig. S15A. Then a HeNe 633 nm laser was used to calibrate the camera intensity to power, which is shown in fig. S15B. After getting the ratio of camera counts to actual power from calibration laser, we can convert the SHG counts in camera to power. Therefore, by dividing the power of 1270 nm pump laser, the SHG conversion efficiency is 5.8×10^{-9} at average power ($Pavg$) of 57 mW. The peak power of the laser is $Pp = Pavg\ f\tau$ = 3.56 kW, where $Pavg=$ 57 mW, f = 80 MHz is the repetition rate of the laser, τ = 200 fs is the pulse duration. The radius of the pump beam is measured to be 11.03 µm, resulting in pump peak intensity of 0.93 $GWcm^{-2}$.

REFERENCES AND NOTES

1. M. V. Rybin, K. L. Koshelev, Z. F. Sadrieva, K. B. Samusev, A. A. Bogdanov, M. F. Limonov, Y. S. Kivshar, High-*Q* supercavity modes in subwavelength dielectric resonators. *Phys. Rev. Lett.* **119**, 243901 (2017).

2. K. Koshelev, G. Favraud, A. Bogdanov, Y. Kivshar, A. Fratalocchi, Nonradiating photonics with resonant dielectric nanostructures. *Nanophotonics* **8**, 725–745 (2019).

3. K. Sergey, Y. Kivshar, Functional meta-optics and nanophotonics governed by Mie resonances. *ACS Photonics* **4**, 2638–2649 (2017).

4. K. Koshelev, S. Lepeshov, M. Liu, A. Bogdanov, Y. Kivshar, Asymmetric metasurfaces with high-*Q* resonances governed by bound states in the continuum. *Phys. Rev. Lett.* **121**, 193903 (2018).

5. K. Koshelev, Y. Tang, K. Li, D.-Y. Choi, G. Li, Y. Kivshar, Nonlinear metasurfaces governed by bound states in the continuum. *ACS Photonics* **6**, 1639–1644 (2019).

6. K. Koshelev, A. Bogdanov, Y. Kivshar, Meta-optics and bound states in the continuum. *Sci. Bull.* **64**, 836–842 (2019).

7. L. Carletti, S. S. Kruk, A. A. Bogdanov, C. De Angelis, Y. Kivshar, High-harmonic generation at the nanoscale boosted by bound states in the continuum. *Phys. Rev. Res.* **1**, 023016 (2019).

8. K. Koshelev, S. Kruk, E. Melik-Gaykazyan, J.-H. Choi, A. Bogdanov, H.-G. Park, Y. Kivshar, Subwavelength dielectric resonators for nonlinear nanophotonics. *Science* **367**, 288–292 (2020).

9. C. Schlickriede, S. S. Kruk, L. Wang, B. Sain, Y. Kivshar, T. Zentgraf, Nonlinear imaging with all-dielectric metasurfaces. *Nano Lett.* **20**, 4370–4376 (2020).

10. G. Grinblat, Y. Li, M. P. Nielsen, R. F. Oulton, S. A. Maier, Efficient third harmonic generation and nonlinear subwavelength imaging at a higher-order anapole mode in a single germanium nanodisk. *ACS Nano* **11**, 953–960 (2017).

11. S. Liu, M. B. Sinclair, S. Saravi, G. A. Keeler, Y. Yang, J. Reno, G. M. Peake, F. Setzpfandt, I. Staude, T. Pertsch, I. Brener, Resonantly enhanced second-harmonic generation using III-V semiconductor all-dielectric metasurfaces. *Nano Lett.* **16**, 5426–5432 (2016).

12. R. Camacho-Morales, M. Rahmani, S. Kruk, L. Wang, L. Xu, D. A. Smirnova, A. S. Solntsev, A. Miroshnichenko, H. H. Tan, F. Karouta, S. Naureen, K. Vora, L. Carletti, C. De Angelis, C. Jagadish, Y. S. Kivshar, D. N. Neshev, Nonlinear generation of vector beams from algaas nanoantennas. *Nano Lett.* **16**, 7191–7197 (2016).

13. F. Yesilkoy, E. R. Arvelo, Y. Jahani, M. Liu, A. Tittl, V. Cevher, Y. Kivshar, H. Altug, Ultrasensitive hyperspectral imaging and biodetection enabled by dielectric metasurfaces. *Nat. Photonics* **13**, 390–396 (2019).

14. V. Kravtsov, E. Khestanova, F. A. Benimetskiy, T. Ivanova, A. K. Samusev, I. S. Sinev, D. Pidgayko, A. M. Mozharov, I. S. Mukhin, M. S. Lozhkin, Y. V. Kapitonov, A. S. Brichkin, V. D. Kulakovskii, I. A. Shelykh, A. I. Tartakovskii, P. M. Walker, M. S. Skolnick, D. N. Krizhanovskii, I. V. Iorsh, Nonlinear polaritons in a monolayer semiconductor coupled to optical bound states in the continuum. *Light Sci. Appl.* **9**, 56 (2020).

15. P. Xie, Z. Liang, T. Jia, D. Li, Y. Chen, P. Chang, H. Zhang, W. Wang, Strong coupling between excitons in a two-dimensional atomic crystal and quasibound states in the continuum in a two-dimensional all-dielectric asymmetric metasurface. *Phys. Rev. B* **104**, 125446 (2021).

16. Y. Xie, Q. Chen, J. Yao, X. Liu, Z. Dong, J. Zhu, Dielectric metasurface evolution from bulk to monolayer by strong coupling of quasi-BICs for second harmonic boosting. *Photonics Res.* **12**, 784–792 (2024).

17. Z. Li, X. Tian, C.-W. Qiu, J. S. Ho, Metasurfaces for bioelectronics and healthcare. *Nat. Electron.* **4**, 382–391 (2021).

18. B. Schwarz, Mapping the world in 3D. *Nat. Photonics* **4**, 429–430 (2010).

19. J. Yu, S. Park, I. Hwang, D. Kim, F. Demmerle, G. Boehm, M. C. Amann, M. A. Belkin, J. Lee, Electrically tunable nonlinear polaritonic metasurface. *Nat. Photonics* **16**, 72–78 (2022).

20. A. Krasnok, M. Tymchenko, A. Alù, Nonlinear metasurfaces: A paradigm shift in nonlinear optics. *Mater. Today* **21**, 8–21 (2018).

21. G. Li, S. Zhang, T. Zentgraf, Nonlinear photonic metasurfaces. *Nat. Rev. Mater.* **2**, 17010 (2017).

22. T. Weber, L. Kühner, L. Sortino, A. Ben Mhenni, N. P. Wilson, J. Kühne, J. J. Finley, S. A. Maier, A. Tittl, Intrinsic strong light-matter coupling with self-hybridized bound states in the continuum in van der Waals metasurfaces. *Nat. Mater.* **22**, 970–976 (2023).

23. S. Das, G. Gupta, K. Majumdar, Layer degree of freedom for excitons in transition metal dichalcogenides. *Phys. Rev. B* **99**, 165411 (2019).

24. A. Arora, M. Drüppel, R. Schmidt, T. Deilmann, R. Schneider, M. R. Molas, P. Marauhn, S. M. de Vasconcellos, M. Potemski, M. Rohlfing, R. Bratschitsch, Interlayer excitons in a bulk van der Waals semiconductor. *Nat. Commun.* **8**, 639 (2017).

25. A. V. Stier, N. P. Wilson, G. Clark, X. Xu, S. A. Crooker, Probing the influence of dielectric environment on excitons in monolayer WSe_2: Insight from high magnetic fields. *Nano Lett.* **16**, 7054–7060 (2016).

26. B. Aslan, M. Deng, T. F. Heinz, Strain tuning of excitons in monolayer WSe_2. *Phys. Rev. B.* **98**, 115308 (2018).

27. J. van de Groep, J.-H. Song, U. Celano, Q. Li, P. G. Kik, M. L. Brongersma, Exciton resonance tuning of an atomically thin lens. *Nat. Photonics* **14**, 426–430 (2020).

28. K. L. Seyler, J. R. Schaibley, P. Gong, P. Rivera, A. M. Jones, S. Wu, J. Yan, D. G. Mandrus, W. Yao, X. Xu, Electrical control of second-harmonic generation in a WSe_2 monolayer transistor. *Nat. Nanotechnol.* **10**, 407–411 (2015).

29. H.-L. Liu, T. Yang, J.-H. Chen, H.-W. Chen, H. Gao, R. Saito, M.-Y. Li, L.-J. Li, Temperature-dependent optical constants of monolayer MoS_2, $MoSe_2$, WS_2, and WSe_2: Spectroscopic ellipsometry and first-principles calculations. *Sci. Rep.* **10**, 15282 (2020).

30. T. Santiago-Cruz, S. D. Gennaro, O. Mitrofanov, S. Addamane, J. Reno, I. Brener, M. V. Chekhova, Resonant metasurfaces for generating complex quantum states. *Science* **377**, 991–995 (2022).

31. G. Wang, X. Marie, I. Gerber, T. Amand, D. Lagarde, L. Bouet, M. Vidal, A. Balocchi, B. Urbaszek, Giant enhancement of the optical second-harmonic emission of WSe_2 monolayers by laser excitation at exciton resonances. *Phys. Rev. Lett.* **114**, 097403 (2015).

32. K.-Q. Lin, S. Bange, J. M. Lupton, Quantum interference in second-harmonic generation from monolayer WSe_2. *Nat. Phys.* **15**, 242–246 (2019).

33. H. Zhou, M. Qin, H. Xu, G. Wei, H. Li, W. Gao, J. Liu, F. Wu, Photonic spin-controlled self-hybridized exciton-polaritons inWS_2 metasurfaces driven by chiral quasibound states in the continuum. *Phys. Rev. B* **109**, 125201 (2024).

34. L. Sortino, A. Gale, L. Kühner, C. Li, J. Biechteler, F. J. Wendisch, M. Kianinia, H. Ren, M. Toth, S. A. Maier, I. Aharonovich, A. Tittl, Optically addressable spin defects coupled to bound states in the continuum metasurfaces. *Nat. Commun.* **15**, 2008 (2024).

35. R. Verre, D. G. Baranov, B. Munkhbat, J. Cuadra, M. Käll, T. Shegai, Transition metal dichalcogenide nanodisks as high-index dielectric Mie nanoresonators. *Nat. Nanotechnol.* **14**, 679–683 (2019).

36. A. Arora, T. Deilmann, P. Marauhn, M. Drüppel, R. Schneider, M. R. Molas, D. Vaclavkova, S. M. de Vasconocellos, M. Rohlfing, M. Potemski, R. Bratschitsch, Valley-contrasting optics of interlayer excitons in Mo- and W-based bulk transition metal dichalcogenides. *Nanoscale* **10**, 15571–15577 (2018).

37. X. Xu, W. Yao, D. Xiao, T. F. Heinz, Spin and pseudospins in layered transition metal dichalcogenides. *Nat. Phys.* **10**, 343–350 (2014).

38. N. Bernhardt, K. Koshelev, S. J. U. White, K. W. C. Meng, J. E. Fröch, S. Kim, T. T. Tran, D.-Y. Choi, Y. Kivshar, A. S. Solntsev, Quasi-BIC resonant enhancement of second-harmonic generation in WS_2 monolayers. *Nano Lett.* **20**, 5309–5314 (2020).

39. M. Nauman, J. Yan, D. de Ceglia, M. Rahmani, K. Z. Kamali, C. De Angelis, A. E. Miroshnichenko, Y. Lu, D. Neshev, Tunable unidirectional nonlinear emission from transition-metal-dichalcogenide metasurfaces. *Nat. Commun.* **12**, 5597 (2021).

40. Z. Liu, Y. Xu, Y. Lin, J. Xiang, T. Feng, Q. Cao, J. Li, S. Lan, J. Liu, High-*Q* quasibound states in the continuum for nonlinear metasurfaces. *Phys. Rev. Lett.* **123**, 253901 (2019).

41. T. Feng, Y. Xu, W. Zhang, A. E. Miroshnichenko, Ideal magnetic dipole scattering. *Phys. Rev. Lett.* **118**, 173901 (2017).

42. A. P. Anthur, H. Zhang, R. Paniagua-Dominguez, D. A. Kalashnikov, S. T. Ha, T. W. W. Maß, A. I. Kuznetsov, L. Krivitsky, Continuous wave second harmonic generation enabled by quasi-bound-states in the continuum on gallium phosphide metasurfaces. *Nano Lett.* **20**, 8745–8751 (2020).

43. E. Mobini, R. Alaee, R. W. Boyd, K. Dolgaleva, Giant asymmetric second-harmonic generation in bianisotropic metasurfaces based on bound states in the continuum. *ACS Photonics* **8**, 3234–3240 (2021).

44. M. R. Shcherbakov, D. N. Neshev, B. Hopkins, A. S. Shorokhov, I. Staude, E. V. Melik-Gaykazyan, M. Decker, A. A. Ezhov, A. E. Miroshnichenko, I. Brener, A. A. Fedyanin, Y. S. Kivshar, Enhanced third-harmonic generation in silicon nanoparticles driven by magnetic response. *Nano Lett.* **14**, 6488–6492 (2014).

45. G. A. Ermolaev, D. V. Grudinin, Y. V. Stebunov, K. V. Voronin, V. G. Kravets, J. Duan, A. B. Mazitov, G. I. Tselikov, A. Bylinkin, D. I. Yakobovsky, S. M. Novikov, D. G. Baranov, A. Y. Nikitin, I. A. Kruglov, T. Shegai, P. A. Gonzalez, A. N. Gregorenko, A. V. Arsenin, K. S. Novoselov, V. S. Volkov, Giant optical anisotropy in transition metal dichalcogenides for next-generation photonics. *Nat. Commun.* **12**, 854 (2021).

46. H. Zhang, B. Abhiraman, Q. Zhang, J. Miao, K. Jo, S. Roccasecca, M. W. Knight, A. R. Davoyan, D. Jariwala, Hybrid exciton-plasmon-polaritons in van der Waals semiconductor gratings. *Nat. Commun.* **11**, 3552 (2020).

47. Y. Zhu, B. Wang, Z. Li, J. Zhang, Y. Tang, J. F. Torres, W. Lipiński, L. Fu, Y. Lu, A high-efficiency wavelength-tunable monolayer led with hybrid continuous-pulsed injection. *Adv. Mater.* **33**, 2101375 (2020).

48. F. J. F. Löchner, A. George, K. Koshelev, T. Bucher, E. Najafidehaghani, A. Fedotova, D.-Y. Choi, T. Pertsch, I. Staude, Y. Kivshar, A. Turchanin, F. Setzpfandt, Hybrid dielectric metasurfaces for enhancing second-harmonic generation in chemical vapor deposition grown MoS_2 monolayers. *ACS Photonics* **8**, 218–227 (2021).

49. P. Hong, L. Xu, M. Rahmani, Dual bound states in the continuum enhanced second harmonic generation with transition metal dichalcogenides monolayer. *Opto-Electron. Adv.* **5**, 200097 (2022).

50. B. Munkhbat, D. G. Baranov, M. Stührenberg, M. Wersäll, A. Bisht, T. Shegai, Self-hybridized exciton-polaritons in multilayers of transition metal dichalcogenides for efficient light absorption. *ACS Photonics* **6**, 139–147 (2019).

51. E. Maggiolini, L. Polimeno, F. Todisco, A. D. Renzo, B. Han, M. D. Giorgi, V. Ardizzone, R. Mastria, A. Cannavale, M. Pugliese, V. Maiorano, G. Gigli, D. Gerace, D. Sanvitto, D. Ballarini, Strongly enhanced light–matter coupling of monolayer WS_2 from a bound state in the continuum. *Nat. Mater.* **22**, 964–969 (2023).

52. T. D. Green, D. G. Baranov, B. Munkhbat, R. Verre, T. Shegai, M. Käll, Optical material anisotropy in high-index transition metal dichalcogenide Mie nanoresonators. *Optica* **7**, 680–686 (2020).

53. Z. Zheng, D. Rocco, H. Ren, O. Sergaeva, Y. Zhang, K. B. Whaley, C. Ying, D. de Ceglia, C. De-Angelis, M. Rahmani, L. Xu, Advances in nonlinear metasurfaces for imaging, quantum, and sensing applications. *Nanophotonics* **12**, 4255–4281 (2023).

54. Y. Kivshar, All-dielectric meta-optics and non-linear nanophotonics. *Natl. Sci. Rev.* **5**, 144–158 (2018).

55. M. A. Weissflog, A. Fedotova, Y. Tang, E. A. Santos, B. Laudert, S. Shinde, F. Abtahi, M. Afsharina, I. P. Perez, S. Ritter, H. Qin, J. Janousek, S. Shradha, I. Staude, S. Saravi, T. Pertsch, F. Setzpfandt, Y. Lu, F. Eilenberger, A tunable transition metal dichalcogenide entangled photon-pair source. *Nat. Commun.* **15**, 7600 (2024).

56. G. Zograf, A. Y. Polyakov, M. Bancerek, T. J. Antosiewicz, B. Küçüköz, T. O. Shegai, Combining ultrahigh index with exceptional nonlinearity in resonant transition metal dichalcogenide nanodisks. *Nat. Photonics* **18**, 751–757 (2024).

57. G. Zograf, B. Küçüköz, Alexander Yu. Polyakov, M. Bancerek, A. V. Agrawal, W. Wieczorek, T. J. Antosiewicz, T. O. Shegai, Ultrathin 3R-MoS_2 metasurfaces with atomically precise edges for efficient nonlinear nanophotonics. arXiv:2410.20960 [physics.optics] (2024).

58. Z. Han, F. Ding, Y. Cai, U. Levy, Significantly enhanced second-harmonic generations with all-dielectric antenna array working in the quasi-bound states in the continuum and excited by linearly polarized plane waves. *Nanophotonics* **10**, 1189–1196 (2021).

59. M. Kjellberg, F. Vennberg, A. P. Ravishankar, S. Anand, Polarization-enabled tuning of anapole resonances in vertically stacked elliptical silicon nanodisks. *Adv. Photonics Res.* **5**, 2400009 (2024).

60. Z. Huang, J. Wang, W. Jia, S. Zhang, C. Zhou, High-*Q* all-dielectric metasurface perfect absorber powered by quasi-bound states in the continuum. *Appl. Phys. Lett.* **125**, 141702 (2024).

61. Y. Li, A. Chernikov, X. Zhang, A. Rigosi, H. M. Hill, A. M. van der Zande, D. A. Chenet, E.-M. Shih, J. Hone, T. F. Heinz, Measurement of the optical dielectric function of monolayer transition-metal dichalcogenides: MoS_2, $MoSe_2$, WS_2 and WSe_2. *Phys. Rev. B* **90**, 205422 (2014).

62. T. J. Kim, V. L. Le, H. T. Nguyen, X. A. Nguyen, Y. D. Kim, Modeling of the optical properties of monolayer WS_2. *J. Korean Phys. Soc.* **77**, 298–302 (2020).
